# Supplementary material for: Structural Mutations in the Organellar Genomes of Valeriana sambucifolia f. dageletiana (Nakai. ex Maekawa) Hara Show Dynamic Gene Transfer
Source: Int J Mol Sci. 2021 Apr 5;22(7):3770. doi: 10.3390/ijms22073770 (PMC8038606; doi:10.3390/ijms22073770)
Supplement: Supplementary file 1 [file ijms-22-03770-s001.pdf]

Supplementary Material

Supplementary table 1. Blast results of non-plastome regions of *V. sambucifolia* f. *dageletiana* plastome

Query	Query Len	Query Loc	Subject	Description	Bit Score	Alignment Len	Percent Identity	E value
NPR_1	273	rbcL-accD (LSC)	NA	NA	NA	NA	NA	NA
NPR_2	62	trnN-trnL (IR)	NA	NA	NA	NA	NA	NA
NPR_3	521	trnN-trnL (IR)	NA	NA	NA	NA	NA	NA
NRP_4	183	ycf1 (SSC)	LR736844.1	Pecten maximus genome assembly, chromosome: 7	58.1	64	79.688	0.000342
			XM_019663827.1	PREDICTED: Hipposideros armiger E3 SUMO-protein ligase RanBP2 (LOC109394058), transcript variant X2, mRNA	64.4	69	82.609	2.30E-06
			XM_019663826.1	PREDICTED: Hipposideros armiger E3 SUMO-protein ligase RanBP2 (LOC109394058), transcript variant X1, mRNA	64.4	69	82.609	2.30E-06
			LR782543.1	Arabidopsis thaliana genome assembly, chromosome: 2	59	56	85.714	9.79E-05
			LR699761.1	Arabidopsis thaliana genome assembly, chromosome: 2	59	56	85.714	9.79E-05
			LR797788.1	Arabidopsis thaliana genome assembly, chromosome: 2	59	56	85.714	9.79E-05
			CP002685.1	Arabidopsis thaliana chromosome 2 sequence	59	56	85.714	9.79E-05
			AC005623.3	Arabidopsis thaliana chromosome 2 clone T20P8 map B68, complete sequence	59	56	85.714	9.79E-05
			LR699756.1	Arabidopsis thaliana genome assembly, chromosome: 2	57.2	55	85.455	0.000342
			CT025531.9	Mouse DNA sequence from clone RP23-280C13 on chromosome 9, complete sequence	57.2	51	88.235	0.000342
			AC157997.2	Mus musculus BAC clone RP24-129N18 from chromosome 9, complete sequence	57.2	51	88.235	0.000342
			LT594789.1	Theobroma cacao genome assembly, chromosome: II	58.1	51	86.275	0.000342
			XM_015521267.1	PREDICTED: Diuraphis noxia uncharacterized LOC107171036 (LOC107171036), mRNA	59	42	90.476	9.79E-05
NPR_5	203	ycf1 (SSC)	NA	NA	NA	NA	NA	NA
NPR_6	121	ycf1 (SSC)	NA	NA	NA	NA	NA	NA
NPR_7	348	ycf1 (SSC)	LR812081.1	Danio rerio genome assembly, chromosome: 19	59.9	324	66.975	0.000208
			LR778259.1	Coregonus sp. 'balchen' genome assembly, chromosome: 7	70.7	321	70.405	1.15E-07
			XM_003233387.1	Trichophyton rubrum CBS 118892 hypothetical protein (TERG_06425) mRNA, complete cds	59.9	97	74.227	0.000208
			LT963424.1	Plasmodium sp. gorilla clade G1 genome assembly, chromosome: 11	64.4	223	66.368	4.89E-06
			LR131460.1	Plasmodium falciparum genome assembly, chromosome: 11	67.1	187	67.914	1.40E-06
			LR131300.1	Plasmodium falciparum genome assembly, chromosome: 11	62.6	187	67.38	1.71E-05

AJ493427.1	Plasmodium falciparum partial mRNA for hypothetical protein (Dd2-3.12 gene) clone Dd2-3.12	67.1	187	67.914	1.40E-06
AJ493427.1	Plasmodium falciparum partial mRNA for hypothetical protein (Dd2-3.12 gene) clone Dd2-3.12	62.6	187	67.38	1.71E-05
LR131412.1	Plasmodium falciparum strain GB4 genome assembly, chromosome: 11	62.6	187	67.38	1.71E-05
LR131477.1	Plasmodium falciparum genome assembly, chromosome: 12	58.1	187	66.845	0.000725
LR131444.1	Plasmodium falciparum genome assembly, chromosome: 11	58.1	187	66.845	0.000725
LR131350.1	Plasmodium falciparum strain HB3 genome assembly, chromosome: 13	58.1	187	66.845	0.000725
LN999945.1	Plasmodium falciparum 3D7 genome assembly, chromosome: 11	58.1	187	66.845	0.000725
XM_001348129.1	Plasmodium falciparum 3D7 ring-infected erythrocyte surface antigen (PF3D7_1149200), partial mRNA	58.1	187	66.845	0.000725
LR131334.1	Plasmodium falciparum strain IT genome assembly, chromosome: 13	60.8	151	68.874	5.95E-05
LR131380.1	Plasmodium falciparum genome assembly, chromosome: 11	60.8	93	74.194	5.95E-05
XM_008819127.1	Plasmodium inui San Antonio 1 hypothetical protein partial mRNA	62.6	44	90.909	1.71E-05
XM_008819127.1	Plasmodium inui San Antonio 1 hypothetical protein partial mRNA	58.1	44	88.636	0.000725
LT841163.1	Plasmodium vivax genome assembly, chromosome: 14	58.1	44	88.636	0.000725
XM_001616968.1	Plasmodium vivax hypothetical protein, conserved (PVX_122275), partial mRNA	58.1	44	88.636	0.000725
LR812521.1	Danio kyathit genome assembly, chromosome: 3	59	299	64.883	0.000208
LN714486.1	TPA_asm: Neospora caninum Liverpool, chromosome chrXI, complete genome	87.8	183	70.492	4.28E-13
XM_003885574.1	Neospora caninum Liverpool conserved hypothetical protein (NCLIV_060200), partial mRNA	87.8	183	70.492	4.28E-13
FR823392.1	Neospora caninum Liverpool complete genome, chromosome XI	87.8	183	70.492	4.28E-13
LN714486.1	TPA_asm: Neospora caninum Liverpool, chromosome chrXI, complete genome	89.7	129	75.194	1.23E-13
XM_003885574.1	Neospora caninum Liverpool conserved hypothetical protein (NCLIV_060200), partial mRNA	89.7	129	75.194	1.23E-13
FR823392.1	Neospora caninum Liverpool complete genome, chromosome XI	89.7	129	75.194	1.23E-13
CP020675.1	Oryzias latipes strain Hd-rR chromosome 11 sequence	65.3	93	75.269	4.89E-06
LR812040.1	Danio rerio genome assembly, chromosome: 3	71.6	287	66.551	3.29E-08
XM_038837876.1	PREDICTED: Tripterygium wilfordii uncharacterized LOC119991531 (LOC119991531), mRNA	62.6	54	85.185	1.71E-05
CP020631.1	Oryzias latipes strain HSOK chromosome 11	71.6	326	65.031	3.29E-08
LR812040.1	Danio rerio genome assembly, chromosome: 3	60.8	333	65.766	5.95E-05
CP020631.1	Oryzias latipes strain HSOK chromosome 11	81.5	287	66.202	6.36E-11
CP020631.1	Oryzias latipes strain HSOK chromosome 11	72.5	287	65.157	3.29E-08
CP020631.1	Oryzias latipes strain HSOK chromosome 11	63.5	287	64.46	1.71E-05
LN714486.1	TPA_asm: Neospora caninum Liverpool, chromosome chrXI, complete genome	80.6	164	70.732	6.36E-11
XM_003885574.1	Neospora caninum Liverpool conserved hypothetical protein (NCLIV_060200), partial mRNA	80.6	164	70.732	6.36E-11
FR823392.1	Neospora caninum Liverpool complete genome, chromosome XI	80.6	164	70.732	6.36E-11
CP020631.1	Oryzias latipes strain HSOK chromosome 11	57.2	131	69.466	0.000725

LR812040.1	Danio rerio genome assembly, chromosome: 3	67.1	198	68.182	1.40E-06
LR131935.1	Cottoperca gobio genome assembly, chromosome: 5	85.1	301	66.445	5.22E-12
OB662152.1	Cyprideis torosa	60.8	309	65.049	5.95E-05
LN714486.1	TPA_asm: Neospora caninum Liverpool, chromosome chrXI, complete genome	86	177	70.621	1.50E-12
XM_003885574.1	Neospora caninum Liverpool conserved hypothetical protein (NCLIV_060200), partial mRNA	86	177	70.621	1.50E-12
FR823392.1	Neospora caninum Liverpool complete genome, chromosome XI	86	177	70.621	1.50E-12
LR812065.1	Danio rerio genome assembly, chromosome: 3	71.6	300	66	3.29E-08
XM_021472248.1	PREDICTED: Danio rerio uncharacterized LOC101885714 (LOC101885714), mRNA	71.6	300	66	3.29E-08
FP326669.2	Zebrafish DNA sequence from clone ZFOS-137B5 in linkage group 3, complete sequence	71.6	300	66	3.29E-08
LR812040.1	Danio rerio genome assembly, chromosome: 3	57.2	288	65.972	0.000725
LR812040.1	Danio rerio genome assembly, chromosome: 3	59.9	287	66.551	0.000208
LR132037.1	Anabas testudineus genome assembly, chromosome: 10	109	299	68.562	1.31E-19
LR597463.1	Sphaeramia orbicularis genome assembly, chromosome: 6	123	304	70.066	5.95E-24
LR594556.1	Streptopelia turtur genome assembly, chromosome: 5	57.2	299	65.886	0.000725
LR989866.1	Autographa gamma genome assembly, chromosome: 17	91.5	284	67.606	3.52E-14
LR812512.1	Danio aesculapii genome assembly, chromosome: 7	58.1	237	70.464	0.000725
LN590699.1	Cyprinus carpio genome assembly common carp genome, scaffold: LG22, chromosome: 22	63.5	230	67.391	1.71E-05
LR812525.1	Danio kyathit genome assembly, chromosome: 7	57.2	300	67.333	0.000725
LR812529.1	Danio kyathit genome assembly, chromosome: 11	156	295	73.559	1.01E-33
LR812494.1	Danio aesculapii genome assembly, chromosome: 22	86.9	290	71.724	1.50E-12
LR812494.1	Danio aesculapii genome assembly, chromosome: 22	84.2	295	70.847	5.22E-12
LR812494.1	Danio aesculapii genome assembly, chromosome: 22	93.3	275	72.364	1.01E-14
XM_001527872.1	Lodderomyces elongisporus NRRL YB-4239 hypothetical protein (LELG_00442) partial mRNA	66.2	106	73.585	1.40E-06
LR812498.1	Danio aesculapii genome assembly, chromosome: 5	82.4	311	69.132	1.82E-11
LR131935.1	Cottoperca gobio genome assembly, chromosome: 5	98.7	298	67.785	2.37E-16
LR131935.1	Cottoperca gobio genome assembly, chromosome: 5	92.4	292	67.466	3.52E-14
LR131935.1	Cottoperca gobio genome assembly, chromosome: 5	66.2	294	66.327	1.40E-06
XM_034133537.1	PREDICTED: Trematomus bernacchii uncharacterized protein LOC793007 homolog (zgc:162331), transcript variant X4, mRNA	223	290	77.241	8.25E-54
XM_034133537.1	PREDICTED: Trematomus bernacchii uncharacterized protein LOC793007 homolog (zgc:162331), transcript variant X4, mRNA	209	290	76.207	1.82E-49
XM_034133537.1	PREDICTED: Trematomus bernacchii uncharacterized protein LOC793007 homolog (zgc:162331), transcript variant X4, mRNA	206	290	75.517	6.35E-49
XM_034133536.1	PREDICTED: Trematomus bernacchii uncharacterized protein LOC793007 homolog (zgc:162331), transcript variant X3, mRNA	223	290	77.241	8.25E-54
XM_034133536.1	PREDICTED: Trematomus bernacchii uncharacterized protein LOC793007 homolog (zgc:162331), transcript variant X3, mRNA	209	290	76.207	1.82E-49

XM_034133536.1	PREDICTED: Trematomus bernacchii uncharacterized protein LOC793007 homolog (zgc:162331), transcript variant X3, mRNA	206	290	75.517	6.35E-49
XM_034133535.1	PREDICTED: Trematomus bernacchii uncharacterized protein LOC793007 homolog (zgc:162331), transcript variant X2, mRNA	223	290	77.241	8.25E-54
XM_034133535.1	PREDICTED: Trematomus bernacchii uncharacterized protein LOC793007 homolog (zgc:162331), transcript variant X2, mRNA	209	290	76.207	1.82E-49
XM_034133535.1	PREDICTED: Trematomus bernacchii uncharacterized protein LOC793007 homolog (zgc:162331), transcript variant X2, mRNA	206	290	75.517	6.35E-49
XM_034133534.1	PREDICTED: Trematomus bernacchii uncharacterized protein LOC793007 homolog (zgc:162331), transcript variant X1, mRNA	223	290	77.241	8.25E-54
XM_034133534.1	PREDICTED: Trematomus bernacchii uncharacterized protein LOC793007 homolog (zgc:162331), transcript variant X1, mRNA	209	290	76.207	1.82E-49
XM_034133534.1	PREDICTED: Trematomus bernacchii uncharacterized protein LOC793007 homolog (zgc:162331), transcript variant X1, mRNA	206	290	75.517	6.35E-49
LR597467.1	Sphaeramia orbicularis genome assembly, chromosome: 10	113	291	69.759	1.08E-20
LR131935.1	Cottoperca gobio genome assembly, chromosome: 5	80.6	297	66.667	6.36E-11
LR597460.1	Sphaeramia orbicularis genome assembly, chromosome: 3	77	297	66.33	7.74E-10
XM_034133537.1	PREDICTED: Trematomus bernacchii uncharacterized protein LOC793007 homolog (zgc:162331), transcript variant X4, mRNA	197	286	75.524	3.29E-46
XM_034133536.1	PREDICTED: Trematomus bernacchii uncharacterized protein LOC793007 homolog (zgc:162331), transcript variant X3, mRNA	197	286	75.524	3.29E-46
XM_034133535.1	PREDICTED: Trematomus bernacchii uncharacterized protein LOC793007 homolog (zgc:162331), transcript variant X2, mRNA	197	286	75.524	3.29E-46
XM_034133534.1	PREDICTED: Trematomus bernacchii uncharacterized protein LOC793007 homolog (zgc:162331), transcript variant X1, mRNA	197	286	75.524	3.29E-46
LR597463.1	Sphaeramia orbicularis genome assembly, chromosome: 6	149	286	72.028	1.49E-31
XM_034133537.1	PREDICTED: Trematomus bernacchii uncharacterized protein LOC793007 homolog (zgc:162331), transcript variant X4, mRNA	223	285	77.544	8.25E-54
XM_034133536.1	PREDICTED: Trematomus bernacchii uncharacterized protein LOC793007 homolog (zgc:162331), transcript variant X3, mRNA	223	285	77.544	8.25E-54
XM_034133535.1	PREDICTED: Trematomus bernacchii uncharacterized protein LOC793007 homolog (zgc:162331), transcript variant X2, mRNA	223	285	77.544	8.25E-54
XM_034133534.1	PREDICTED: Trematomus bernacchii uncharacterized protein LOC793007 homolog (zgc:162331), transcript variant X1, mRNA	223	285	77.544	8.25E-54
LR722980.1	Thalassophryne amazonica genome assembly, chromosome: 15	77	316	68.354	7.74E-10
XM_034133537.1	PREDICTED: Trematomus bernacchii uncharacterized protein LOC793007 homolog (zgc:162331), transcript variant X4, mRNA	200	280	76.071	9.42E-47
XM_034133536.1	PREDICTED: Trematomus bernacchii uncharacterized protein LOC793007 homolog (zgc:162331), transcript variant X3, mRNA	200	280	76.071	9.42E-47
XM_034133535.1	PREDICTED: Trematomus bernacchii uncharacterized protein LOC793007 homolog (zgc:162331), transcript variant X2, mRNA	200	280	76.071	9.42E-47

XM_034133534.1	PREDICTED: Trematomus bernacchii uncharacterized protein LOC793007 homolog (zgc:162331), transcript variant X1, mRNA	200	280	76.071	9.42E-47
LR584427.1	Salmo trutta genome assembly, chromosome: 19	92.4	278	69.065	3.52E-14
LR597469.1	Sphaeramia orbicularis genome assembly, chromosome: 12	68	294	69.728	4.01E-07
LR584427.1	Salmo trutta genome assembly, chromosome: 19	85.1	263	68.821	5.22E-12
LR584406.1	Salmo trutta genome assembly, chromosome: 6	72.5	281	67.26	3.29E-08
LR597467.1	Sphaeramia orbicularis genome assembly, chromosome: 10	95.1	244	69.672	2.89E-15
LR597467.1	Sphaeramia orbicularis genome assembly, chromosome: 10	90.6	213	70.892	1.23E-13
LR597467.1	Sphaeramia orbicularis genome assembly, chromosome: 10	59.9	140	70.714	0.000208
OB787669.1	Cyprideis torosa	60.8	141	73.759	5.95E-05
XM_034133537.1	PREDICTED: Trematomus bernacchii uncharacterized protein LOC793007 homolog (zgc:162331), transcript variant X4, mRNA	94.2	101	81.188	1.01E-14
XM_034133536.1	PREDICTED: Trematomus bernacchii uncharacterized protein LOC793007 homolog (zgc:162331), transcript variant X3, mRNA	94.2	101	81.188	1.01E-14
XM_034133535.1	PREDICTED: Trematomus bernacchii uncharacterized protein LOC793007 homolog (zgc:162331), transcript variant X2, mRNA	94.2	101	81.188	1.01E-14
XM_034133534.1	PREDICTED: Trematomus bernacchii uncharacterized protein LOC793007 homolog (zgc:162331), transcript variant X1, mRNA	94.2	101	81.188	1.01E-14
LR812593.1	Danio rerio strain Nadia (NA) genome assembly, chromosome: 25	63.5	309	68.285	1.71E-05
LR597476.1	Sphaeramia orbicularis genome assembly, chromosome: 19	57.2	256	64.062	0.000725
LN714486.1	TPA_asm: Neospora caninum Liverpool, chromosome chrXI, complete genome	81.5	202	68.812	6.36E-11
XM_003885574.1	Neospora caninum Liverpool conserved hypothetical protein (NCLIV_060200), partial mRNA	81.5	202	68.812	6.36E-11
FR823392.1	Neospora caninum Liverpool complete genome, chromosome XI	81.5	202	68.812	6.36E-11
LR990951.1	Noctua fimbriata genome assembly, chromosome: 30	62.6	294	64.966	1.71E-05
XM_034133537.1	PREDICTED: Trematomus bernacchii uncharacterized protein LOC793007 homolog (zgc:162331), transcript variant X4, mRNA	218	291	76.976	3.51E-52
XM_034133537.1	PREDICTED: Trematomus bernacchii uncharacterized protein LOC793007 homolog (zgc:162331), transcript variant X4, mRNA	214	288	76.736	4.28E-51
XM_034133537.1	PREDICTED: Trematomus bernacchii uncharacterized protein LOC793007 homolog (zgc:162331), transcript variant X4, mRNA	205	288	76.042	2.22E-48
XM_034133537.1	PREDICTED: Trematomus bernacchii uncharacterized protein LOC793007 homolog (zgc:162331), transcript variant X4, mRNA	201	288	75.694	2.70E-47
XM_034133536.1	PREDICTED: Trematomus bernacchii uncharacterized protein LOC793007 homolog (zgc:162331), transcript variant X3, mRNA	218	291	76.976	3.51E-52
XM_034133536.1	PREDICTED: Trematomus bernacchii uncharacterized protein LOC793007 homolog (zgc:162331), transcript variant X3, mRNA	214	288	76.736	4.28E-51
XM_034133536.1	PREDICTED: Trematomus bernacchii uncharacterized protein LOC793007 homolog (zgc:162331), transcript variant X3, mRNA	205	288	76.042	2.22E-48
XM_034133536.1	PREDICTED: Trematomus bernacchii uncharacterized protein LOC793007 homolog (zgc:162331), transcript variant X3, mRNA	201	288	75.694	2.70E-47

XM_034133535.1	PREDICTED: Trematomus bernacchii uncharacterized protein LOC793007 homolog (zgc:162331), transcript variant X2, mRNA	218	291	76.976	3.51E-52
XM_034133535.1	PREDICTED: Trematomus bernacchii uncharacterized protein LOC793007 homolog (zgc:162331), transcript variant X2, mRNA	214	288	76.736	4.28E-51
XM_034133535.1	PREDICTED: Trematomus bernacchii uncharacterized protein LOC793007 homolog (zgc:162331), transcript variant X2, mRNA	205	288	76.042	2.22E-48
XM_034133535.1	PREDICTED: Trematomus bernacchii uncharacterized protein LOC793007 homolog (zgc:162331), transcript variant X2, mRNA	201	288	75.694	2.70E-47
XM_034133534.1	PREDICTED: Trematomus bernacchii uncharacterized protein LOC793007 homolog (zgc:162331), transcript variant X1, mRNA	218	291	76.976	3.51E-52
XM_034133534.1	PREDICTED: Trematomus bernacchii uncharacterized protein LOC793007 homolog (zgc:162331), transcript variant X1, mRNA	214	288	76.736	4.28E-51
XM_034133534.1	PREDICTED: Trematomus bernacchii uncharacterized protein LOC793007 homolog (zgc:162331), transcript variant X1, mRNA	205	288	76.042	2.22E-48
XM_034133534.1	PREDICTED: Trematomus bernacchii uncharacterized protein LOC793007 homolog (zgc:162331), transcript variant X1, mRNA	201	288	75.694	2.70E-47
LR597458.1	Sphaeramia orbicularis genome assembly, chromosome: 1	124	290	71.724	5.95E-24
LR597458.1	Sphaeramia orbicularis genome assembly, chromosome: 1	122	299	69.9	2.08E-23
LR597458.1	Sphaeramia orbicularis genome assembly, chromosome: 1	113	305	71.148	1.08E-20
LR597458.1	Sphaeramia orbicularis genome assembly, chromosome: 1	150	291	73.883	4.28E-32
LR597458.1	Sphaeramia orbicularis genome assembly, chromosome: 1	149	287	72.125	1.49E-31
LR597458.1	Sphaeramia orbicularis genome assembly, chromosome: 1	142	293	73.72	2.22E-29
LR597458.1	Sphaeramia orbicularis genome assembly, chromosome: 1	132	299	72.575	1.15E-26
LR597458.1	Sphaeramia orbicularis genome assembly, chromosome: 1	123	295	71.864	5.95E-24
XM_034133537.1	PREDICTED: Trematomus bernacchii uncharacterized protein LOC793007 homolog (zgc:162331), transcript variant X4, mRNA	225	284	77.817	2.36E-54
XM_034133537.1	PREDICTED: Trematomus bernacchii uncharacterized protein LOC793007 homolog (zgc:162331), transcript variant X4, mRNA	221	284	77.465	2.88E-53
XM_034133537.1	PREDICTED: Trematomus bernacchii uncharacterized protein LOC793007 homolog (zgc:162331), transcript variant X4, mRNA	217	296	76.014	3.51E-52
XM_034133537.1	PREDICTED: Trematomus bernacchii uncharacterized protein LOC793007 homolog (zgc:162331), transcript variant X4, mRNA	216	284	77.113	1.23E-51
XM_034133537.1	PREDICTED: Trematomus bernacchii uncharacterized protein LOC793007 homolog (zgc:162331), transcript variant X4, mRNA	212	284	76.761	1.49E-50
XM_034133537.1	PREDICTED: Trematomus bernacchii uncharacterized protein LOC793007 homolog (zgc:162331), transcript variant X4, mRNA	204	296	75.338	7.73E-48
XM_034133537.1	PREDICTED: Trematomus bernacchii uncharacterized protein LOC793007 homolog (zgc:162331), transcript variant X4, mRNA	198	284	75.704	3.29E-46
XM_034133536.1	PREDICTED: Trematomus bernacchii uncharacterized protein LOC793007 homolog (zgc:162331), transcript variant X3, mRNA	225	284	77.817	2.36E-54

XM_034133536.1	PREDICTED: Trematomus bernacchii uncharacterized protein LOC793007 homolog (zgc:162331), transcript variant X3, mRNA	221	284	77.465	2.88E-53
XM_034133536.1	PREDICTED: Trematomus bernacchii uncharacterized protein LOC793007 homolog (zgc:162331), transcript variant X3, mRNA	217	296	76.014	3.51E-52
XM_034133536.1	PREDICTED: Trematomus bernacchii uncharacterized protein LOC793007 homolog (zgc:162331), transcript variant X3, mRNA	216	284	77.113	1.23E-51
XM_034133536.1	PREDICTED: Trematomus bernacchii uncharacterized protein LOC793007 homolog (zgc:162331), transcript variant X3, mRNA	212	284	76.761	1.49E-50
XM_034133536.1	PREDICTED: Trematomus bernacchii uncharacterized protein LOC793007 homolog (zgc:162331), transcript variant X3, mRNA	204	296	75.338	7.73E-48
XM_034133536.1	PREDICTED: Trematomus bernacchii uncharacterized protein LOC793007 homolog (zgc:162331), transcript variant X3, mRNA	198	284	75.704	3.29E-46
XM_034133535.1	PREDICTED: Trematomus bernacchii uncharacterized protein LOC793007 homolog (zgc:162331), transcript variant X2, mRNA	225	284	77.817	2.36E-54
XM_034133535.1	PREDICTED: Trematomus bernacchii uncharacterized protein LOC793007 homolog (zgc:162331), transcript variant X2, mRNA	221	284	77.465	2.88E-53
XM_034133535.1	PREDICTED: Trematomus bernacchii uncharacterized protein LOC793007 homolog (zgc:162331), transcript variant X2, mRNA	217	296	76.014	3.51E-52
XM_034133535.1	PREDICTED: Trematomus bernacchii uncharacterized protein LOC793007 homolog (zgc:162331), transcript variant X2, mRNA	216	284	77.113	1.23E-51
XM_034133535.1	PREDICTED: Trematomus bernacchii uncharacterized protein LOC793007 homolog (zgc:162331), transcript variant X2, mRNA	212	284	76.761	1.49E-50
XM_034133535.1	PREDICTED: Trematomus bernacchii uncharacterized protein LOC793007 homolog (zgc:162331), transcript variant X2, mRNA	204	296	75.338	7.73E-48
XM_034133535.1	PREDICTED: Trematomus bernacchii uncharacterized protein LOC793007 homolog (zgc:162331), transcript variant X2, mRNA	198	284	75.704	3.29E-46
XM_034133534.1	PREDICTED: Trematomus bernacchii uncharacterized protein LOC793007 homolog (zgc:162331), transcript variant X1, mRNA	225	284	77.817	2.36E-54
XM_034133534.1	PREDICTED: Trematomus bernacchii uncharacterized protein LOC793007 homolog (zgc:162331), transcript variant X1, mRNA	221	284	77.465	2.88E-53
XM_034133534.1	PREDICTED: Trematomus bernacchii uncharacterized protein LOC793007 homolog (zgc:162331), transcript variant X1, mRNA	217	296	76.014	3.51E-52
XM_034133534.1	PREDICTED: Trematomus bernacchii uncharacterized protein LOC793007 homolog (zgc:162331), transcript variant X1, mRNA	216	284	77.113	1.23E-51
XM_034133534.1	PREDICTED: Trematomus bernacchii uncharacterized protein LOC793007 homolog (zgc:162331), transcript variant X1, mRNA	212	284	76.761	1.49E-50
XM_034133534.1	PREDICTED: Trematomus bernacchii uncharacterized protein LOC793007 homolog (zgc:162331), transcript variant X1, mRNA	204	296	75.338	7.73E-48
XM_034133534.1	PREDICTED: Trematomus bernacchii uncharacterized protein LOC793007 homolog (zgc:162331), transcript variant X1, mRNA	198	284	75.704	3.29E-46
XM_010775385.1	PREDICTED: Notothenia coriiceps mediator of RNA polymerase II transcription subunit 15-like (LOC104949106), mRNA	216	284	77.113	1.23E-51

XM_010775385.1	PREDICTED: Notothenia coriiceps mediator of RNA polymerase II transcription subunit 15-like (LOC104949106), mRNA	212	284	76.761	1.49E-50
XM_010775385.1	PREDICTED: Notothenia coriiceps mediator of RNA polymerase II transcription subunit 15-like (LOC104949106), mRNA	207	284	76.408	6.35E-49
XM_010775385.1	PREDICTED: Notothenia coriiceps mediator of RNA polymerase II transcription subunit 15-like (LOC104949106), mRNA	203	284	76.056	7.73E-48
XM_010775385.1	PREDICTED: Notothenia coriiceps mediator of RNA polymerase II transcription subunit 15-like (LOC104949106), mRNA	193	287	75.261	1.40E-44
XM_010775385.1	PREDICTED: Notothenia coriiceps mediator of RNA polymerase II transcription subunit 15-like (LOC104949106), mRNA	170	287	73.519	4.57E-38
XM_034225939.1	PREDICTED: Gymnodraco acuticeps uncharacterized protein LOC793007 homolog (zgc:162331), mRNA	203	284	76.056	7.73E-48
XM_034225939.1	PREDICTED: Gymnodraco acuticeps uncharacterized protein LOC793007 homolog (zgc:162331), mRNA	189	284	75	1.70E-43
XM_034133537.1	PREDICTED: Trematomus bernacchii uncharacterized protein LOC793007 homolog (zgc:162331), transcript variant X4, mRNA	223	286	77.622	8.25E-54
XM_034133537.1	PREDICTED: Trematomus bernacchii uncharacterized protein LOC793007 homolog (zgc:162331), transcript variant X4, mRNA	214	283	77.032	4.28E-51
XM_034133537.1	PREDICTED: Trematomus bernacchii uncharacterized protein LOC793007 homolog (zgc:162331), transcript variant X4, mRNA	210	283	76.678	5.21E-50
XM_034133537.1	PREDICTED: Trematomus bernacchii uncharacterized protein LOC793007 homolog (zgc:162331), transcript variant X4, mRNA	205	283	76.325	2.22E-48
XM_034133537.1	PREDICTED: Trematomus bernacchii uncharacterized protein LOC793007 homolog (zgc:162331), transcript variant X4, mRNA	201	283	75.972	2.70E-47
XM_034133536.1	PREDICTED: Trematomus bernacchii uncharacterized protein LOC793007 homolog (zgc:162331), transcript variant X3, mRNA	223	286	77.622	8.25E-54
XM_034133536.1	PREDICTED: Trematomus bernacchii uncharacterized protein LOC793007 homolog (zgc:162331), transcript variant X3, mRNA	214	283	77.032	4.28E-51
XM_034133536.1	PREDICTED: Trematomus bernacchii uncharacterized protein LOC793007 homolog (zgc:162331), transcript variant X3, mRNA	210	283	76.678	5.21E-50
XM_034133536.1	PREDICTED: Trematomus bernacchii uncharacterized protein LOC793007 homolog (zgc:162331), transcript variant X3, mRNA	205	283	76.325	2.22E-48
XM_034133536.1	PREDICTED: Trematomus bernacchii uncharacterized protein LOC793007 homolog (zgc:162331), transcript variant X3, mRNA	201	283	75.972	2.70E-47
XM_034133535.1	PREDICTED: Trematomus bernacchii uncharacterized protein LOC793007 homolog (zgc:162331), transcript variant X2, mRNA	223	286	77.622	8.25E-54
XM_034133535.1	PREDICTED: Trematomus bernacchii uncharacterized protein LOC793007 homolog (zgc:162331), transcript variant X2, mRNA	214	283	77.032	4.28E-51
XM_034133535.1	PREDICTED: Trematomus bernacchii uncharacterized protein LOC793007 homolog (zgc:162331), transcript variant X2, mRNA	210	283	76.678	5.21E-50
XM_034133535.1	PREDICTED: Trematomus bernacchii uncharacterized protein LOC793007 homolog (zgc:162331), transcript variant X2, mRNA	205	283	76.325	2.22E-48

XM_034133535.1	PREDICTED: Trematomus bernacchii uncharacterized protein LOC793007 homolog (zgc:162331), transcript variant X2, mRNA	201	283	75.972	2.70E-47
XM_034133534.1	PREDICTED: Trematomus bernacchii uncharacterized protein LOC793007 homolog (zgc:162331), transcript variant X1, mRNA	223	286	77.622	8.25E-54
XM_034133534.1	PREDICTED: Trematomus bernacchii uncharacterized protein LOC793007 homolog (zgc:162331), transcript variant X1, mRNA	214	283	77.032	4.28E-51
XM_034133534.1	PREDICTED: Trematomus bernacchii uncharacterized protein LOC793007 homolog (zgc:162331), transcript variant X1, mRNA	210	283	76.678	5.21E-50
XM_034133534.1	PREDICTED: Trematomus bernacchii uncharacterized protein LOC793007 homolog (zgc:162331), transcript variant X1, mRNA	205	283	76.325	2.22E-48
XM_034133534.1	PREDICTED: Trematomus bernacchii uncharacterized protein LOC793007 homolog (zgc:162331), transcript variant X1, mRNA	201	283	75.972	2.70E-47
LR597458.1	Sphaeramia orbicularis genome assembly, chromosome: 1	73.4	310	69.032	9.44E-09
LR990951.1	Noctua fimbriata genome assembly, chromosome: 30	69.8	284	65.493	1.15E-07
XM_034133537.1	PREDICTED: Trematomus bernacchii uncharacterized protein LOC793007 homolog (zgc:162331), transcript variant X4, mRNA	201	278	76.259	2.70E-47
XM_034133536.1	PREDICTED: Trematomus bernacchii uncharacterized protein LOC793007 homolog (zgc:162331), transcript variant X3, mRNA	201	278	76.259	2.70E-47
XM_034133535.1	PREDICTED: Trematomus bernacchii uncharacterized protein LOC793007 homolog (zgc:162331), transcript variant X2, mRNA	201	278	76.259	2.70E-47
XM_034133534.1	PREDICTED: Trematomus bernacchii uncharacterized protein LOC793007 homolog (zgc:162331), transcript variant X1, mRNA	201	278	76.259	2.70E-47
XM_010775385.1	PREDICTED: Notothenia coriiceps mediator of RNA polymerase II transcription subunit 15-like (LOC104949106), mRNA	210	278	76.978	5.21E-50
XM_010775385.1	PREDICTED: Notothenia coriiceps mediator of RNA polymerase II transcription subunit 15-like (LOC104949106), mRNA	178	281	74.377	3.08E-40
XM_034133537.1	PREDICTED: Trematomus bernacchii uncharacterized protein LOC793007 homolog (zgc:162331), transcript variant X4, mRNA	212	274	77.372	1.49E-50
XM_034133536.1	PREDICTED: Trematomus bernacchii uncharacterized protein LOC793007 homolog (zgc:162331), transcript variant X3, mRNA	212	274	77.372	1.49E-50
XM_034133535.1	PREDICTED: Trematomus bernacchii uncharacterized protein LOC793007 homolog (zgc:162331), transcript variant X2, mRNA	212	274	77.372	1.49E-50
XM_034133534.1	PREDICTED: Trematomus bernacchii uncharacterized protein LOC793007 homolog (zgc:162331), transcript variant X1, mRNA	212	274	77.372	1.49E-50
LR990951.1	Noctua fimbriata genome assembly, chromosome: 30	64.4	273	65.568	4.89E-06
LR597458.1	Sphaeramia orbicularis genome assembly, chromosome: 1	126	270	72.963	1.70E-24
LR597458.1	Sphaeramia orbicularis genome assembly, chromosome: 1	84.2	309	67.961	5.22E-12
LR597458.1	Sphaeramia orbicularis genome assembly, chromosome: 1	79.7	309	67.314	2.22E-10
LR597458.1	Sphaeramia orbicularis genome assembly, chromosome: 1	72.5	305	67.541	3.29E-08
LR597458.1	Sphaeramia orbicularis genome assembly, chromosome: 1	70.7	293	67.918	1.15E-07

XM_034133537.1	PREDICTED: Trematomus bernacchii uncharacterized protein LOC793007 homolog (zgc:162331), transcript variant X4, mRNA	182	255	76.078	2.53E-41
XM_034133536.1	PREDICTED: Trematomus bernacchii uncharacterized protein LOC793007 homolog (zgc:162331), transcript variant X3, mRNA	182	255	76.078	2.53E-41
XM_034133535.1	PREDICTED: Trematomus bernacchii uncharacterized protein LOC793007 homolog (zgc:162331), transcript variant X2, mRNA	182	255	76.078	2.53E-41
XM_034133534.1	PREDICTED: Trematomus bernacchii uncharacterized protein LOC793007 homolog (zgc:162331), transcript variant X1, mRNA	182	255	76.078	2.53E-41
XM_010775385.1	PREDICTED: Notothenia coriiceps mediator of RNA polymerase II transcription subunit 15-like (LOC104949106), mRNA	196	255	77.255	1.15E-45
XM_034225939.1	PREDICTED: Gymnodraco acuticeps uncharacterized protein LOC793007 homolog (zgc:162331), mRNA	173	255	75.294	1.31E-38
LR812522.1	Danio kyathit genome assembly, chromosome: 4	64.4	238	68.487	4.89E-06
LR597458.1	Sphaeramia orbicularis genome assembly, chromosome: 1	69.8	249	69.076	1.15E-07
XM_034133537.1	PREDICTED: Trematomus bernacchii uncharacterized protein LOC793007 homolog (zgc:162331), transcript variant X4, mRNA	132	182	76.374	4.01E-26
XM_034133536.1	PREDICTED: Trematomus bernacchii uncharacterized protein LOC793007 homolog (zgc:162331), transcript variant X3, mRNA	132	182	76.374	4.01E-26
XM_034133535.1	PREDICTED: Trematomus bernacchii uncharacterized protein LOC793007 homolog (zgc:162331), transcript variant X2, mRNA	132	182	76.374	4.01E-26
XM_034133534.1	PREDICTED: Trematomus bernacchii uncharacterized protein LOC793007 homolog (zgc:162331), transcript variant X1, mRNA	132	182	76.374	4.01E-26
XM_010775385.1	PREDICTED: Notothenia coriiceps mediator of RNA polymerase II transcription subunit 15-like (LOC104949106), mRNA	136	182	76.923	9.43E-28
LR597458.1	Sphaeramia orbicularis genome assembly, chromosome: 1	83.3	180	70.556	1.82E-11
XM_034133537.1	PREDICTED: Trematomus bernacchii uncharacterized protein LOC793007 homolog (zgc:162331), transcript variant X4, mRNA	139	171	78.363	2.70E-28
XM_034133536.1	PREDICTED: Trematomus bernacchii uncharacterized protein LOC793007 homolog (zgc:162331), transcript variant X3, mRNA	139	171	78.363	2.70E-28
XM_034133535.1	PREDICTED: Trematomus bernacchii uncharacterized protein LOC793007 homolog (zgc:162331), transcript variant X2, mRNA	139	171	78.363	2.70E-28
XM_034133534.1	PREDICTED: Trematomus bernacchii uncharacterized protein LOC793007 homolog (zgc:162331), transcript variant X1, mRNA	139	171	78.363	2.70E-28
XM_010775385.1	PREDICTED: Notothenia coriiceps mediator of RNA polymerase II transcription subunit 15-like (LOC104949106), mRNA	143	171	78.947	6.35E-30
XM_034133537.1	PREDICTED: Trematomus bernacchii uncharacterized protein LOC793007 homolog (zgc:162331), transcript variant X4, mRNA	83.3	110	77.273	1.82E-11
XM_034133536.1	PREDICTED: Trematomus bernacchii uncharacterized protein LOC793007 homolog (zgc:162331), transcript variant X3, mRNA	83.3	110	77.273	1.82E-11
XM_034133535.1	PREDICTED: Trematomus bernacchii uncharacterized protein LOC793007 homolog (zgc:162331), transcript variant X2, mRNA	83.3	110	77.273	1.82E-11

XM_034133534.1	PREDICTED: Trematomus bernacchii uncharacterized protein LOC793007 homolog (zgc:162331), transcript variant X1, mRNA	83.3	110	77.273	1.82E-11
XM_010775385.1	PREDICTED: Notothenia coriiceps mediator of RNA polymerase II transcription subunit 15-like (LOC104949106), mRNA	83.3	110	77.273	1.82E-11
XM_010775385.1	PREDICTED: Notothenia coriiceps mediator of RNA polymerase II transcription subunit 15-like (LOC104949106), mRNA	86	99	79.798	1.50E-12
LR812073.1	Danio rerio genome assembly, chromosome: 11	102	303	69.307	1.94E-17
LR812532.1	Danio kyathit genome assembly, chromosome: 14	62.6	244	65.574	1.71E-05
LR812532.1	Danio kyathit genome assembly, chromosome: 14	58.1	244	65.164	0.000725
LR812065.1	Danio rerio genome assembly, chromosome: 3	63.5	231	67.532	1.71E-05
XM_021472248.1	PREDICTED: Danio rerio uncharacterized LOC101885714 (LOC101885714), mRNA	63.5	231	67.532	1.71E-05
FP326669.2	Zebrafish DNA sequence from clone ZFOS-137B5 in linkage group 3, complete sequence	63.5	231	67.532	1.71E-05
LR597457.1	Salarias fasciatus genome assembly, chromosome: 23	61.7	204	71.569	5.95E-05
LR778269.1	Coregonus sp. 'balchen' genome assembly, chromosome: 17	59	173	67.63	0.000208
LR812106.1	Erithacus rubecula genome assembly, chromosome: 4	57.2	307	67.101	0.000725
LR812540.1	Danio kyathit genome assembly, chromosome: 22	59.9	291	69.072	0.000208
LR597463.1	Sphaeramia orbicularis genome assembly, chromosome: 6	62.6	276	69.928	1.71E-05
LR812065.1	Danio rerio genome assembly, chromosome: 3	61.7	165	69.091	5.95E-05
XM_021472248.1	PREDICTED: Danio rerio uncharacterized LOC101885714 (LOC101885714), mRNA	61.7	165	69.091	5.95E-05
FP326669.2	Zebrafish DNA sequence from clone ZFOS-137B5 in linkage group 3, complete sequence	61.7	165	69.091	5.95E-05
LR131935.1	Cottoperca gobio genome assembly, chromosome: 5	104	293	68.259	5.57E-18
LR131935.1	Cottoperca gobio genome assembly, chromosome: 5	95.1	293	67.577	2.89E-15
LR131935.1	Cottoperca gobio genome assembly, chromosome: 5	81.5	289	67.474	6.36E-11
LR131935.1	Cottoperca gobio genome assembly, chromosome: 5	68	290	66.207	4.01E-07
OE843916.1	5_Tge_b3v08	60.8	283	64.664	5.95E-05
LR812065.1	Danio rerio genome assembly, chromosome: 3	63.5	294	66.327	1.71E-05
XM_021472248.1	PREDICTED: Danio rerio uncharacterized LOC101885714 (LOC101885714), mRNA	63.5	294	66.327	1.71E-05
FP326669.2	Zebrafish DNA sequence from clone ZFOS-137B5 in linkage group 3, complete sequence	63.5	294	66.327	1.71E-05
LR131935.1	Cottoperca gobio genome assembly, chromosome: 5	85.1	278	66.906	5.22E-12
CP020674.1	Oryzias latipes strain Hd-rR chromosome 10 sequence	93.3	282	67.73	1.01E-14
LR131935.1	Cottoperca gobio genome assembly, chromosome: 5	77	274	67.153	7.74E-10
LR744059.1	Scyliorhinus canicula genome assembly, chromosome: 30	67.1	272	66.176	1.40E-06
LR812040.1	Danio rerio genome assembly, chromosome: 3	83.3	283	68.905	1.82E-11
LR812065.1	Danio rerio genome assembly, chromosome: 3	68.9	285	65.263	4.01E-07
XM_021472248.1	PREDICTED: Danio rerio uncharacterized LOC101885714 (LOC101885714), mRNA	68.9	285	65.263	4.01E-07
FP326669.2	Zebrafish DNA sequence from clone ZFOS-137B5 in linkage group 3, complete sequence	68.9	285	65.263	4.01E-07
CP026260.1	Scophthalmus maximus chromosome 18	108	273	73.993	4.57E-19

LR812506.1	Danio aesculapii genome assembly, chromosome: 11	80.6	308	67.208	6.36E-11
LR584432.1	Salmo trutta genome assembly, chromosome: 30	126	290	70.69	1.70E-24
LR584432.1	Salmo trutta genome assembly, chromosome: 30	123	288	70.139	2.08E-23
LR722984.1	Thalassophryne amazonica genome assembly, chromosome: 19	75.2	306	66.993	2.70E-09
LR722984.1	Thalassophryne amazonica genome assembly, chromosome: 19	68.9	292	67.123	4.01E-07
LR597463.1	Sphaeramia orbicularis genome assembly, chromosome: 6	144	291	70.79	6.35E-30
LR597463.1	Sphaeramia orbicularis genome assembly, chromosome: 6	134	291	70.447	3.29E-27
LR597463.1	Sphaeramia orbicularis genome assembly, chromosome: 6	133	297	70.707	1.15E-26
LR597463.1	Sphaeramia orbicularis genome assembly, chromosome: 6	127	291	69.759	4.88E-25
LR597463.1	Sphaeramia orbicularis genome assembly, chromosome: 6	122	297	70.707	2.08E-23
LR597463.1	Sphaeramia orbicularis genome assembly, chromosome: 6	122	297	71.044	2.08E-23
LR597463.1	Sphaeramia orbicularis genome assembly, chromosome: 6	120	285	69.825	7.25E-23
LR597463.1	Sphaeramia orbicularis genome assembly, chromosome: 6	117	292	69.521	8.83E-22
LR597463.1	Sphaeramia orbicularis genome assembly, chromosome: 6	113	292	69.863	1.08E-20
LR597463.1	Sphaeramia orbicularis genome assembly, chromosome: 6	111	291	69.072	3.75E-20
LR597463.1	Sphaeramia orbicularis genome assembly, chromosome: 6	111	297	68.687	3.75E-20
LR597463.1	Sphaeramia orbicularis genome assembly, chromosome: 6	103	289	69.204	1.94E-17
LR597463.1	Sphaeramia orbicularis genome assembly, chromosome: 6	77	304	67.434	7.74E-10
LR584432.1	Salmo trutta genome assembly, chromosome: 30	139	296	70.27	2.70E-28
LR597461.1	Sphaeramia orbicularis genome assembly, chromosome: 4	113	290	69.31	1.08E-20
LR131935.1	Cottoperca gobio genome assembly, chromosome: 5	100	287	67.944	6.79E-17
LR131935.1	Cottoperca gobio genome assembly, chromosome: 5	97.8	292	67.808	8.27E-16
LR131935.1	Cottoperca gobio genome assembly, chromosome: 5	96	293	67.577	2.89E-15
LR131935.1	Cottoperca gobio genome assembly, chromosome: 5	93.3	286	67.832	1.01E-14
LR131935.1	Cottoperca gobio genome assembly, chromosome: 5	85.1	293	68.259	5.22E-12
LR131935.1	Cottoperca gobio genome assembly, chromosome: 5	77.9	295	68.136	7.74E-10
LR131935.1	Cottoperca gobio genome assembly, chromosome: 5	61.7	294	65.646	5.95E-05
LN591021.1	Cyprinus carpio genome assembly common carp genome, scaffold 000001169	67.1	317	70.347	1.40E-06
XM_034133537.1	PREDICTED: Trematomus bernacchii uncharacterized protein LOC793007 homolog (zgc:162331), transcript variant X4, mRNA	218	283	77.032	3.51E-52
XM_034133537.1	PREDICTED: Trematomus bernacchii uncharacterized protein LOC793007 homolog (zgc:162331), transcript variant X4, mRNA	205	283	75.972	2.22E-48
XM_034133536.1	PREDICTED: Trematomus bernacchii uncharacterized protein LOC793007 homolog (zgc:162331), transcript variant X3, mRNA	218	283	77.032	3.51E-52
XM_034133536.1	PREDICTED: Trematomus bernacchii uncharacterized protein LOC793007 homolog (zgc:162331), transcript variant X3, mRNA	205	283	75.972	2.22E-48
XM_034133535.1	PREDICTED: Trematomus bernacchii uncharacterized protein LOC793007 homolog (zgc:162331), transcript variant X2, mRNA	218	283	77.032	3.51E-52
XM_034133535.1	PREDICTED: Trematomus bernacchii uncharacterized protein LOC793007 homolog (zgc:162331), transcript variant X2, mRNA	205	283	75.972	2.22E-48

XM_034133534.1	PREDICTED: Trematomus bernacchii uncharacterized protein LOC793007 homolog (zgc:162331), transcript variant X1, mRNA	218	283	77.032	3.51E-52
XM_034133534.1	PREDICTED: Trematomus bernacchii uncharacterized protein LOC793007 homolog (zgc:162331), transcript variant X1, mRNA	205	283	75.972	2.22E-48
LR131916.1	Cottoperca gobio genome assembly, chromosome: 1	134	292	72.26	3.29E-27
LR131935.1	Cottoperca gobio genome assembly, chromosome: 5	89.7	288	67.708	1.23E-13
LR738552.1	Neostethus bicornis genome assembly, chromosome: 22	72.5	289	66.436	3.29E-08
LR812554.1	Danio rerio strain Cooch Behar (CB) genome assembly, chromosome: 11	62.6	306	66.993	1.71E-05
LR597458.1	Sphaeramia orbicularis genome assembly, chromosome: 1	136	291	71.821	9.43E-28
LR597458.1	Sphaeramia orbicularis genome assembly, chromosome: 1	120	304	70.395	7.25E-23
LR132037.1	Anabas testudineus genome assembly, chromosome: 10	109	282	69.149	1.31E-19
LR131935.1	Cottoperca gobio genome assembly, chromosome: 5	80.6	290	66.897	6.36E-11
LR597463.1	Sphaeramia orbicularis genome assembly, chromosome: 6	114	281	69.395	3.08E-21
LR812523.1	Danio kyathit genome assembly, chromosome: 5	71.6	308	66.558	3.29E-08
LR812045.1	Danio rerio genome assembly, chromosome: 8	77	300	67	7.74E-10
LR812045.1	Danio rerio genome assembly, chromosome: 8	74.3	316	66.456	9.44E-09
LR597458.1	Sphaeramia orbicularis genome assembly, chromosome: 1	159	287	75.261	2.88E-34
LR597458.1	Sphaeramia orbicularis genome assembly, chromosome: 1	143	292	73.973	6.35E-30
XM_034133537.1	PREDICTED: Trematomus bernacchii uncharacterized protein LOC793007 homolog (zgc:162331), transcript variant X4, mRNA	229	279	78.136	1.94E-55
XM_034133537.1	PREDICTED: Trematomus bernacchii uncharacterized protein LOC793007 homolog (zgc:162331), transcript variant X4, mRNA	224	279	77.778	2.36E-54
XM_034133537.1	PREDICTED: Trematomus bernacchii uncharacterized protein LOC793007 homolog (zgc:162331), transcript variant X4, mRNA	211	279	76.703	5.21E-50
XM_034133537.1	PREDICTED: Trematomus bernacchii uncharacterized protein LOC793007 homolog (zgc:162331), transcript variant X4, mRNA	202	279	75.986	2.70E-47
XM_034133537.1	PREDICTED: Trematomus bernacchii uncharacterized protein LOC793007 homolog (zgc:162331), transcript variant X4, mRNA	179	279	74.194	8.82E-41
XM_034133536.1	PREDICTED: Trematomus bernacchii uncharacterized protein LOC793007 homolog (zgc:162331), transcript variant X3, mRNA	229	279	78.136	1.94E-55
XM_034133536.1	PREDICTED: Trematomus bernacchii uncharacterized protein LOC793007 homolog (zgc:162331), transcript variant X3, mRNA	224	279	77.778	2.36E-54
XM_034133536.1	PREDICTED: Trematomus bernacchii uncharacterized protein LOC793007 homolog (zgc:162331), transcript variant X3, mRNA	211	279	76.703	5.21E-50
XM_034133536.1	PREDICTED: Trematomus bernacchii uncharacterized protein LOC793007 homolog (zgc:162331), transcript variant X3, mRNA	202	279	75.986	2.70E-47
XM_034133536.1	PREDICTED: Trematomus bernacchii uncharacterized protein LOC793007 homolog (zgc:162331), transcript variant X3, mRNA	179	279	74.194	8.82E-41
XM_034133535.1	PREDICTED: Trematomus bernacchii uncharacterized protein LOC793007 homolog (zgc:162331), transcript variant X2, mRNA	229	279	78.136	1.94E-55

XM_034133535.1	PREDICTED: Trematomus bernacchii uncharacterized protein LOC793007 homolog (zgc:162331), transcript variant X2, mRNA	224	279	77.778	2.36E-54
XM_034133535.1	PREDICTED: Trematomus bernacchii uncharacterized protein LOC793007 homolog (zgc:162331), transcript variant X2, mRNA	211	279	76.703	5.21E-50
XM_034133535.1	PREDICTED: Trematomus bernacchii uncharacterized protein LOC793007 homolog (zgc:162331), transcript variant X2, mRNA	202	279	75.986	2.70E-47
XM_034133535.1	PREDICTED: Trematomus bernacchii uncharacterized protein LOC793007 homolog (zgc:162331), transcript variant X2, mRNA	179	279	74.194	8.82E-41
XM_034133534.1	PREDICTED: Trematomus bernacchii uncharacterized protein LOC793007 homolog (zgc:162331), transcript variant X1, mRNA	229	279	78.136	1.94E-55
XM_034133534.1	PREDICTED: Trematomus bernacchii uncharacterized protein LOC793007 homolog (zgc:162331), transcript variant X1, mRNA	224	279	77.778	2.36E-54
XM_034133534.1	PREDICTED: Trematomus bernacchii uncharacterized protein LOC793007 homolog (zgc:162331), transcript variant X1, mRNA	211	279	76.703	5.21E-50
XM_034133534.1	PREDICTED: Trematomus bernacchii uncharacterized protein LOC793007 homolog (zgc:162331), transcript variant X1, mRNA	202	279	75.986	2.70E-47
XM_034133534.1	PREDICTED: Trematomus bernacchii uncharacterized protein LOC793007 homolog (zgc:162331), transcript variant X1, mRNA	179	279	74.194	8.82E-41
XM_010775385.1	PREDICTED: Notothenia coriiceps mediator of RNA polymerase II transcription subunit 15-like (LOC104949106), mRNA	215	279	77.061	1.23E-51
XM_034225939.1	PREDICTED: Gymnodraco acuticeps uncharacterized protein LOC793007 homolog (zgc:162331), mRNA	193	279	75.269	1.40E-44
XM_034225939.1	PREDICTED: Gymnodraco acuticeps uncharacterized protein LOC793007 homolog (zgc:162331), mRNA	170	279	73.477	4.57E-38
XM_029876167.1	PREDICTED: Aedes albopictus RNA-binding protein 25-like (LOC115268134), mRNA	170	279	73.477	4.57E-38
XM_029876167.1	PREDICTED: Aedes albopictus RNA-binding protein 25-like (LOC115268134), mRNA	161	279	72.76	2.37E-35
LR584445.1	Salmo trutta genome assembly, chromosome: 2	170	287	75.61	4.57E-38
LR584445.1	Salmo trutta genome assembly, chromosome: 2	167	286	74.825	5.57E-37
LR584445.1	Salmo trutta genome assembly, chromosome: 2	163	286	74.825	6.78E-36
LR597478.1	Sphaeramia orbicularis genome assembly, chromosome: 21	166	279	73.118	1.94E-36
LR597463.1	Sphaeramia orbicularis genome assembly, chromosome: 6	150	279	72.401	1.49E-31
LR597463.1	Sphaeramia orbicularis genome assembly, chromosome: 6	138	285	70.526	2.70E-28
LR597463.1	Sphaeramia orbicularis genome assembly, chromosome: 6	136	291	71.134	9.43E-28
LR597463.1	Sphaeramia orbicularis genome assembly, chromosome: 6	129	279	71.326	1.40E-25
LR597463.1	Sphaeramia orbicularis genome assembly, chromosome: 6	128	286	70.629	4.88E-25
LR597463.1	Sphaeramia orbicularis genome assembly, chromosome: 6	127	286	70.28	4.88E-25
LR597463.1	Sphaeramia orbicularis genome assembly, chromosome: 6	127	279	70.609	4.88E-25
LR597463.1	Sphaeramia orbicularis genome assembly, chromosome: 6	116	285	70.175	8.83E-22
LR597463.1	Sphaeramia orbicularis genome assembly, chromosome: 6	113	285	69.474	1.08E-20
LR597463.1	Sphaeramia orbicularis genome assembly, chromosome: 6	113	293	69.283	1.08E-20
LR597463.1	Sphaeramia orbicularis genome assembly, chromosome: 6	109	279	68.817	1.31E-19

LR597463.1	Sphaeramia orbicularis genome assembly, chromosome: 6	104	293	69.966	5.57E-18
LR597463.1	Sphaeramia orbicularis genome assembly, chromosome: 6	89.7	286	67.832	1.23E-13
LR584432.1	Salmo trutta genome assembly, chromosome: 30	124	280	70.714	5.95E-24
LR584432.1	Salmo trutta genome assembly, chromosome: 30	123	279	69.892	2.08E-23
LR131935.1	Cottoperca gobio genome assembly, chromosome: 5	87.8	285	67.018	4.28E-13
LR131935.1	Cottoperca gobio genome assembly, chromosome: 5	79.7	282	67.73	2.22E-10
LR131935.1	Cottoperca gobio genome assembly, chromosome: 5	75.2	286	66.783	2.70E-09
LR131935.1	Cottoperca gobio genome assembly, chromosome: 5	68.9	283	67.138	4.01E-07
XM_027158426.1	PREDICTED: Tachysurus fulvidraco splicing factor U2af large subunit A-like (LOC113650229), mRNA	92.4	280	67.5	3.52E-14
LR131971.1	Parambassis ranga genome assembly, chromosome: 20	101	315	67.619	6.79E-17
LR131971.1	Parambassis ranga genome assembly, chromosome: 20	96	315	67.619	2.89E-15
LR131971.1	Parambassis ranga genome assembly, chromosome: 20	92.4	315	66.984	3.52E-14
LR131971.1	Parambassis ranga genome assembly, chromosome: 20	89.7	311	67.203	1.23E-13
LR697110.1	Chanos chanos genome assembly, chromosome: 5	94.2	279	67.384	1.01E-14
LR812506.1	Danio aesculapii genome assembly, chromosome: 11	80.6	303	69.307	6.36E-11
LR812498.1	Danio aesculapii genome assembly, chromosome: 5	86	304	69.737	1.50E-12
LR633949.1	Gadus morhua genome assembly, chromosome: 7	70.7	289	69.55	1.15E-07
LR812045.1	Danio rerio genome assembly, chromosome: 8	83.3	305	67.541	1.82E-11
LR597472.1	Sphaeramia orbicularis genome assembly, chromosome: 15	81.5	285	67.719	6.36E-11
LR597472.1	Sphaeramia orbicularis genome assembly, chromosome: 15	73.4	294	68.367	9.44E-09
LR812525.1	Danio kyathit genome assembly, chromosome: 7	79.7	289	68.858	2.22E-10
LN591021.1	Cyprinus carpio genome assembly common carp genome, scaffold 000001169	59.9	304	70.724	0.000208
XM_034133537.1	PREDICTED: Trematomus bernacchii uncharacterized protein LOC793007 homolog (zgc:162331), transcript variant X4, mRNA	196	278	75.54	1.15E-45
XM_034133536.1	PREDICTED: Trematomus bernacchii uncharacterized protein LOC793007 homolog (zgc:162331), transcript variant X3, mRNA	196	278	75.54	1.15E-45
XM_034133535.1	PREDICTED: Trematomus bernacchii uncharacterized protein LOC793007 homolog (zgc:162331), transcript variant X2, mRNA	196	278	75.54	1.15E-45
XM_034133534.1	PREDICTED: Trematomus bernacchii uncharacterized protein LOC793007 homolog (zgc:162331), transcript variant X1, mRNA	196	278	75.54	1.15E-45
LR597478.1	Sphaeramia orbicularis genome assembly, chromosome: 21	146	283	71.378	1.82E-30
LR812070.1	Danio rerio genome assembly, chromosome: 8	105	300	69.667	1.60E-18
LR812070.1	Danio rerio genome assembly, chromosome: 8	77.9	290	67.241	7.74E-10
XM_029876167.1	PREDICTED: Aedes albopictus RNA-binding protein 25-like (LOC115268134), mRNA	167	277	73.285	5.57E-37
XM_029876167.1	PREDICTED: Aedes albopictus RNA-binding protein 25-like (LOC115268134), mRNA	162	277	72.924	2.37E-35
LR597463.1	Sphaeramia orbicularis genome assembly, chromosome: 6	126	277	71.48	1.70E-24
LR812073.1	Danio rerio genome assembly, chromosome: 11	121	298	71.141	7.25E-23
LR812070.1	Danio rerio genome assembly, chromosome: 8	91.5	293	68.601	3.52E-14
LR812069.1	Danio rerio genome assembly, chromosome: 7	96	289	70.934	2.89E-15

LR131935.1	Cottoperca gobio genome assembly, chromosome: 5	81.5	287	68.641	6.36E-11
LR131935.1	Cottoperca gobio genome assembly, chromosome: 5	77.9	280	67.5	7.74E-10
LR131935.1	Cottoperca gobio genome assembly, chromosome: 5	77	291	67.698	7.74E-10
LR131935.1	Cottoperca gobio genome assembly, chromosome: 5	68	285	66.316	4.01E-07
LR812498.1	Danio aesculapii genome assembly, chromosome: 5	86.9	293	71.331	1.50E-12
LR812063.1	Danio rerio genome assembly, chromosome: 1	113	274	69.343	1.08E-20
LR812063.1	Danio rerio genome assembly, chromosome: 1	102	282	68.085	1.94E-17
CP026255.1	Scophthalmus maximus chromosome 13	94.2	303	70.297	1.01E-14
XM_027158426.1	PREDICTED: Tachysurus fulvidraco splicing factor U2af large subunit A-like (LOC113650229), mRNA	102	273	68.498	1.94E-17
XM_027158426.1	PREDICTED: Tachysurus fulvidraco splicing factor U2af large subunit A-like (LOC113650229), mRNA	97.8	273	68.132	8.27E-16
CR385022.10	Zebrafish DNA sequence from clone CH211-63C12 in linkage group 23, complete sequence	86.9	287	73.171	1.50E-12
XM_027158426.1	PREDICTED: Tachysurus fulvidraco splicing factor U2af large subunit A-like (LOC113650229), mRNA	96	272	68.015	2.89E-15
LR597463.1	Sphaeramia orbicularis genome assembly, chromosome: 6	127	272	71.691	4.88E-25
LR597463.1	Sphaeramia orbicularis genome assembly, chromosome: 6	118	272	70.588	2.53E-22
LR597463.1	Sphaeramia orbicularis genome assembly, chromosome: 6	109	285	70.877	1.31E-19
LR597463.1	Sphaeramia orbicularis genome assembly, chromosome: 6	109	276	68.841	1.31E-19
LR597463.1	Sphaeramia orbicularis genome assembly, chromosome: 6	107	272	69.853	4.57E-19
LR597461.1	Sphaeramia orbicularis genome assembly, chromosome: 4	108	275	69.455	4.57E-19
LR131935.1	Cottoperca gobio genome assembly, chromosome: 5	77.9	283	66.784	7.74E-10
LR697111.1	Chanos chanos genome assembly, chromosome: 6	60.8	278	65.827	5.95E-05
XM_034133537.1	PREDICTED: Trematomus bernacchii uncharacterized protein LOC793007 homolog (zgc:162331), transcript variant X4, mRNA	211	269	77.323	5.21E-50
XM_034133536.1	PREDICTED: Trematomus bernacchii uncharacterized protein LOC793007 homolog (zgc:162331), transcript variant X3, mRNA	211	269	77.323	5.21E-50
XM_034133535.1	PREDICTED: Trematomus bernacchii uncharacterized protein LOC793007 homolog (zgc:162331), transcript variant X2, mRNA	211	269	77.323	5.21E-50
XM_034133534.1	PREDICTED: Trematomus bernacchii uncharacterized protein LOC793007 homolog (zgc:162331), transcript variant X1, mRNA	211	269	77.323	5.21E-50
LR597478.1	Sphaeramia orbicularis genome assembly, chromosome: 21	125	269	70.26	1.70E-24
LR812494.1	Danio aesculapii genome assembly, chromosome: 22	106	282	74.468	1.60E-18
LR812494.1	Danio aesculapii genome assembly, chromosome: 22	103	281	74.021	1.94E-17
LR812506.1	Danio aesculapii genome assembly, chromosome: 11	93.3	285	69.123	1.01E-14
LR812506.1	Danio aesculapii genome assembly, chromosome: 11	71.6	295	68.136	3.29E-08
LR812506.1	Danio aesculapii genome assembly, chromosome: 11	62.6	279	66.667	1.71E-05
LR812523.1	Danio kyathit genome assembly, chromosome: 5	85.1	277	69.675	5.22E-12
LR597472.1	Sphaeramia orbicularis genome assembly, chromosome: 15	77.9	274	67.518	7.74E-10

LR812538.1	Danio kyathit genome assembly, chromosome: 20	75.2	282	71.631	2.70E-09
LR597469.1	Sphaeramia orbicularis genome assembly, chromosome: 12	71.6	276	72.464	3.29E-08
LR812522.1	Danio kyathit genome assembly, chromosome: 4	75.2	284	68.31	2.70E-09
LR778265.1	Coregonus sp. 'balchen' genome assembly, chromosome: 13	70.7	285	71.579	1.15E-07
LR778265.1	Coregonus sp. 'balchen' genome assembly, chromosome: 13	66.2	277	70.397	1.40E-06
LR778259.1	Coregonus sp. 'balchen' genome assembly, chromosome: 7	64.4	278	70.504	4.89E-06
LR597470.1	Sphaeramia orbicularis genome assembly, chromosome: 13	68	279	68.459	4.01E-07
LR812065.1	Danio rerio genome assembly, chromosome: 3	65.3	286	66.434	4.89E-06
XM_021472248.1	PREDICTED: Danio rerio uncharacterized LOC101885714 (LOC101885714), mRNA	65.3	286	66.434	4.89E-06
FP326669.2	Zebrafish DNA sequence from clone ZFOS-137B5 in linkage group 3, complete sequence	65.3	286	66.434	4.89E-06
LR736844.1	Pecten maximus genome assembly, chromosome: 7	62.6	302	67.55	1.71E-05
LR812065.1	Danio rerio genome assembly, chromosome: 3	57.2	266	65.414	0.000725
LR131935.1	Cottoperca gobio genome assembly, chromosome: 5	82.4	262	67.939	1.82E-11
XM_034225939.1	PREDICTED: Gymnodraco acuticeps uncharacterized protein LOC793007 homolog (zgc:162331), mRNA	164	253	74.308	6.78E-36
LR131999.1	Gouania willdenowi genome assembly, chromosome: 13	121	255	70.98	7.25E-23
LR131935.1	Cottoperca gobio genome assembly, chromosome: 5	89.7	253	67.984	1.23E-13
LR131935.1	Cottoperca gobio genome assembly, chromosome: 5	84.2	253	67.984	5.22E-12
LR131971.1	Parambassis ranga genome assembly, chromosome: 20	95.1	281	68.327	2.89E-15
LR597472.1	Sphaeramia orbicularis genome assembly, chromosome: 15	77	258	68.217	7.74E-10
LR990609.1	Phalera bucephala genome assembly, chromosome: Z	60.8	253	65.217	5.95E-05
LR535816.1	Denticeps clupeoides genome assembly, chromosome: 4	58.1	257	67.315	0.000725
LR131935.1	Cottoperca gobio genome assembly, chromosome: 5	83.3	251	68.127	1.82E-11
LR131935.1	Cottoperca gobio genome assembly, chromosome: 5	81.5	249	67.871	6.36E-11
LR697110.1	Chanos chanos genome assembly, chromosome: 5	93.3	246	68.293	1.01E-14
LR697110.1	Chanos chanos genome assembly, chromosome: 5	88.7	246	67.886	4.28E-13
LR738542.1	Neostethus bicornis genome assembly, chromosome: 1	97.8	248	70.565	8.27E-16
LR812040.1	Danio rerio genome assembly, chromosome: 3	59	246	65.854	0.000208
OE840970.1	5_Tge_b3v08	60.8	260	67.308	5.95E-05
LR812065.1	Danio rerio genome assembly, chromosome: 3	62.6	254	67.323	1.71E-05
LR812546.1	Danio rerio strain Cooch Behar (CB) genome assembly, chromosome: 3	75.2	255	68.235	2.70E-09
BX908774.13	Zebrafish DNA sequence from clone CH211-103A8 in linkage group 3, complete sequence	62.6	254	67.323	1.71E-05
BX005166.14	Zebrafish DNA sequence from clone CH211-137K18 in linkage group 3, complete sequence	62.6	254	67.323	1.71E-05
LR131935.1	Cottoperca gobio genome assembly, chromosome: 5	62.6	244	67.213	1.71E-05
LR697110.1	Chanos chanos genome assembly, chromosome: 5	90.6	237	68.354	1.23E-13
LR744059.1	Scyliorhinus canicula genome assembly, chromosome: 30	77	234	67.949	7.74E-10
LR584437.1	Salmo trutta genome assembly, chromosome: 21	63.5	252	67.063	1.71E-05

LR812506.1	Danio aesculapii genome assembly, chromosome: 11	61.7	243	67.901	5.95E-05
LR812529.1	Danio kyathit genome assembly, chromosome: 11	83.3	228	68.86	1.82E-11
CP026255.1	Scophthalmus maximus chromosome 13	68	240	68.75	4.01E-07
LR132009.2	Betta splendens genome assembly, chromosome: 16	69.8	222	68.919	1.15E-07
LR990292.1	Apotomis turbidana genome assembly, chromosome: 12	67.1	222	67.117	1.40E-06
LR990988.1	Mamestra brassicae genome assembly, chromosome: 1	83.3	222	68.919	1.82E-11
LR736844.1	Pecten maximus genome assembly, chromosome: 7	68.9	257	68.093	4.01E-07
LR131916.1	Cottoperca gobio genome assembly, chromosome: 1	103	231	70.996	1.94E-17
LR597463.1	Sphaeramia orbicularis genome assembly, chromosome: 6	92.4	205	70.244	3.52E-14
LR812506.1	Danio aesculapii genome assembly, chromosome: 11	65.3	209	67.943	4.89E-06
LR633947.1	Gadus morhua genome assembly, chromosome: 5	74.3	216	68.519	9.44E-09
CP026260.1	Scophthalmus maximus chromosome 18	75.2	197	73.604	2.70E-09
LR738542.1	Neostethus bicornis genome assembly, chromosome: 1	77	189	70.37	7.74E-10
LR584412.1	Salmo trutta genome assembly, chromosome: 24	57.2	181	71.271	0.000725
XM_034225939.1	PREDICTED: Gymnodraco acuticeps uncharacterized protein LOC793007 homolog (zgc:162331), mRNA	112	169	74.556	3.75E-20
LR597478.1	Sphaeramia orbicularis genome assembly, chromosome: 21	76.1	169	69.822	2.70E-09
LR584443.1	Salmo trutta genome assembly, chromosome: 40	73.4	169	71.006	9.44E-09
LR778263.1	Coregonus sp. 'balchen' genome assembly, chromosome: 11	70.7	166	69.277	1.15E-07
LR697110.1	Chanos chanos genome assembly, chromosome: 5	64.4	165	68.485	4.89E-06
LR584426.1	Salmo trutta genome assembly, chromosome: 17	94.2	164	72.561	1.01E-14
XM_027158426.1	PREDICTED: Tachysurus fulvidraco splicing factor U2af large subunit A-like (LOC113650229), mRNA	74.3	163	69.939	9.44E-09
LR697110.1	Chanos chanos genome assembly, chromosome: 5	66.2	161	68.944	1.40E-06
LR633945.1	Gadus morhua genome assembly, chromosome: 3	62.6	169	71.598	1.71E-05
LR584432.1	Salmo trutta genome assembly, chromosome: 30	84.2	161	73.913	5.22E-12
LR990988.1	Mamestra brassicae genome assembly, chromosome: 1	62.6	149	69.128	1.71E-05
LR131916.1	Cottoperca gobio genome assembly, chromosome: 1	81.5	152	73.684	6.36E-11
XM_026453704.2	PREDICTED: Piliocolobus tephrosceles probable ATP-dependent helicase PF08_0048 (LOC113224587), mRNA	69.8	133	71.429	1.15E-07
XM_026453702.2	PREDICTED: Piliocolobus tephrosceles probable ATP-dependent helicase PF08_0048 (LOC113224585), mRNA	69.8	133	71.429	1.15E-07
XM_026453701.2	PREDICTED: Piliocolobus tephrosceles serine/threonine-protein kinase fray2-like (LOC113224584), partial mRNA	69.8	133	71.429	1.15E-07
XM_026453703.1	PREDICTED: Piliocolobus tephrosceles serine/threonine-protein kinase fray2-like (LOC113224586), partial mRNA	69.8	133	71.429	1.15E-07
LR738551.1	Neostethus bicornis genome assembly, chromosome: 11	63.5	125	72.8	1.71E-05
LR778269.1	Coregonus sp. 'balchen' genome assembly, chromosome: 17	62.6	119	71.429	1.71E-05
LR597463.1	Sphaeramia orbicularis genome assembly, chromosome: 6	72.5	114	73.684	3.29E-08
LR131916.1	Cottoperca gobio genome assembly, chromosome: 1	61.7	106	76.415	5.95E-05

LR761660.1	Aphantopus hyperantus genome assembly, chromosome: 13	59	97	73.196	0.000208
LR778263.1	Coregonus sp. 'balchen' genome assembly, chromosome: 11	62.6	94	74.468	1.71E-05
LR812544.1	Danio rerio strain Cooch Behar (CB) genome assembly, chromosome: 1	78.8	93	78.495	2.22E-10
XM_038360266.1	PREDICTED: Zerene cesonia activated Cdc42 kinase-like (LOC119835447), mRNA	78.8	88	79.545	2.22E-10
LR812529.1	Danio kyathit genome assembly, chromosome: 11	129	282	70.567	1.40E-25
LR812529.1	Danio kyathit genome assembly, chromosome: 11	148	287	73.519	5.21E-31
LR597478.1	Sphaeramia orbicularis genome assembly, chromosome: 21	127	278	70.863	4.88E-25
LR131928.1	Cottoperca gobio genome assembly, chromosome: 20	151	280	72.5	4.28E-32
LR131928.1	Cottoperca gobio genome assembly, chromosome: 20	150	278	71.942	4.28E-32
LR131928.1	Cottoperca gobio genome assembly, chromosome: 20	144	279	71.685	6.35E-30
LR131928.1	Cottoperca gobio genome assembly, chromosome: 20	141	278	71.223	2.22E-29
LR131928.1	Cottoperca gobio genome assembly, chromosome: 20	137	278	70.863	9.43E-28
LR812065.1	Danio rerio genome assembly, chromosome: 3	69.8	288	67.361	1.15E-07
LR131983.1	Gouania willdenowi genome assembly, chromosome: 14	76.1	318	67.925	2.70E-09
LR812496.1	Danio aesculapii genome assembly, chromosome: 17	61.7	293	68.259	5.95E-05
LR812496.1	Danio aesculapii genome assembly, chromosome: 17	58.1	292	67.466	0.000725
LR597476.1	Sphaeramia orbicularis genome assembly, chromosome: 19	58.1	306	63.725	0.000725
LR131928.1	Cottoperca gobio genome assembly, chromosome: 20	132	275	70.545	4.01E-26
LR584426.1	Salmo trutta genome assembly, chromosome: 17	126	274	70.438	1.70E-24
LR131983.1	Gouania willdenowi genome assembly, chromosome: 14	60.8	305	66.557	5.95E-05
LR131928.1	Cottoperca gobio genome assembly, chromosome: 20	135	272	70.956	3.29E-27
LR131928.1	Cottoperca gobio genome assembly, chromosome: 20	139	269	71.375	2.70E-28
LR778277.1	Coregonus sp. 'balchen' genome assembly, chromosome: 25	65.3	289	69.55	4.89E-06
LR131928.1	Cottoperca gobio genome assembly, chromosome: 20	142	251	72.51	2.22E-29
LR131928.1	Cottoperca gobio genome assembly, chromosome: 20	138	251	72.112	2.70E-28
LR584426.1	Salmo trutta genome assembly, chromosome: 17	109	235	70.213	1.31E-19
LR722980.1	Thalassophryne amazonica genome assembly, chromosome: 15	67.1	210	69.524	1.40E-06
LR778263.1	Coregonus sp. 'balchen' genome assembly, chromosome: 11	84.2	201	69.154	5.22E-12
LR131928.1	Cottoperca gobio genome assembly, chromosome: 20	101	188	71.809	6.79E-17
LR131928.1	Cottoperca gobio genome assembly, chromosome: 20	86	167	71.257	1.50E-12
LR597479.1	Sphaeramia orbicularis genome assembly, chromosome: 22	66.2	164	68.902	1.40E-06
LR597463.1	Sphaeramia orbicularis genome assembly, chromosome: 6	121	289	70.242	7.25E-23
LR597463.1	Sphaeramia orbicularis genome assembly, chromosome: 6	114	289	69.204	3.08E-21
LR597463.1	Sphaeramia orbicularis genome assembly, chromosome: 6	109	313	69.01	1.31E-19
LR597463.1	Sphaeramia orbicularis genome assembly, chromosome: 6	103	307	69.381	1.94E-17
LR597463.1	Sphaeramia orbicularis genome assembly, chromosome: 6	100	301	69.103	6.79E-17
LR597463.1	Sphaeramia orbicularis genome assembly, chromosome: 6	99.6	295	68.136	2.37E-16
LR131935.1	Cottoperca gobio genome assembly, chromosome: 5	92.4	291	67.354	3.52E-14
LR131935.1	Cottoperca gobio genome assembly, chromosome: 5	78.8	289	66.782	2.22E-10

LR990951.1	Noctua fimbriata genome assembly, chromosome: 30	63.5	287	65.157	1.71E-05
LR736842.1	Pecten maximus genome assembly, chromosome: 5	59	293	66.894	0.000208
LR131935.1	Cottoperca gobio genome assembly, chromosome: 5	86.9	289	68.166	1.50E-12
CP020639.1	Oryzias latipes strain HSOK chromosome 19	68	279	65.233	4.01E-07
CP020639.1	Oryzias latipes strain HSOK chromosome 19	63.5	279	64.875	1.71E-05
OD568792.1	4_Tbi_b3v08	71.6	311	67.524	3.29E-08
XM_034225939.1	PREDICTED: Gymnodraco acuticeps uncharacterized protein LOC793007 homolog (zgc:162331), mRNA	194	277	75.451	4.00E-45
LR597478.1	Sphaeramia orbicularis genome assembly, chromosome: 21	145	294	70.748	1.82E-30
LR597463.1	Sphaeramia orbicularis genome assembly, chromosome: 6	128	277	70.758	4.88E-25
LR597463.1	Sphaeramia orbicularis genome assembly, chromosome: 6	127	277	70.036	4.88E-25
LR597463.1	Sphaeramia orbicularis genome assembly, chromosome: 6	118	278	69.784	2.53E-22
LR597463.1	Sphaeramia orbicularis genome assembly, chromosome: 6	118	283	69.965	2.53E-22
LR597463.1	Sphaeramia orbicularis genome assembly, chromosome: 6	114	291	70.79	3.08E-21
LR597463.1	Sphaeramia orbicularis genome assembly, chromosome: 6	113	278	69.424	1.08E-20
LR597463.1	Sphaeramia orbicularis genome assembly, chromosome: 6	112	278	69.065	3.75E-20
CP068262.1	Homo sapiens isolate CHM13 chromosome 16	58.1	289	65.744	0.000725
AP023476.1	Homo sapiens DNA, chromosome 16, nearly complete genome	58.1	289	65.744	0.000725
CP034494.1	Eukaryotic synthetic construct chromosome 16	62.6	297	65.32	1.71E-05
LR722978.1	Thalassophryne amazonica genome assembly, chromosome: 13	72.5	293	66.553	3.29E-08
AC092341.5	Homo sapiens chromosome 16 clone RP11-260K13, complete sequence	62.6	297	65.32	1.71E-05
LR597463.1	Sphaeramia orbicularis genome assembly, chromosome: 6	116	276	69.203	8.83E-22
LR594556.1	Streptopelia turtur genome assembly, chromosome: 5	62.6	292	66.781	1.71E-05
LR597463.1	Sphaeramia orbicularis genome assembly, chromosome: 6	123	281	70.107	2.08E-23
LR738552.1	Neostethus bicornis genome assembly, chromosome: 22	92.4	281	68.327	3.52E-14
LR597475.1	Sphaeramia orbicularis genome assembly, chromosome: 18	68	288	67.014	4.01E-07
XM_034225939.1	PREDICTED: Gymnodraco acuticeps uncharacterized protein LOC793007 homolog (zgc:162331), mRNA	160	271	73.063	8.26E-35
CP020788.1	Oryzias latipes strain HNI chromosome 10	110	271	70.111	1.31E-19
CP020674.1	Oryzias latipes strain Hd-rR chromosome 10 sequence	80.6	275	66.909	6.36E-11
CP020674.1	Oryzias latipes strain Hd-rR chromosome 10 sequence	77.9	279	67.025	7.74E-10
CP020674.1	Oryzias latipes strain Hd-rR chromosome 10 sequence	73.4	275	66.545	9.44E-09
LR597463.1	Sphaeramia orbicularis genome assembly, chromosome: 6	147	268	72.015	5.21E-31
LR597463.1	Sphaeramia orbicularis genome assembly, chromosome: 6	131	268	70.522	4.01E-26
LR597461.1	Sphaeramia orbicularis genome assembly, chromosome: 4	113	277	70.036	1.08E-20
AC092341.5	Homo sapiens chromosome 16 clone RP11-260K13, complete sequence	58.1	284	65.141	0.000725
CU929147.5	Zebrafish DNA sequence from clone CH73-194O9 in linkage group 3, complete sequence	63.5	260	66.154	1.71E-05
LR584443.1	Salmo trutta genome assembly, chromosome: 40	95.1	251	69.323	2.89E-15
CP020674.1	Oryzias latipes strain Hd-rR chromosome 10 sequence	86	255	67.843	1.50E-12

CP020788.1	Oryzias latipes strain HNI chromosome 10	108	256	70.312	4.57E-19
CP020674.1	Oryzias latipes strain Hd-rR chromosome 10 sequence	86	254	68.11	1.50E-12
CP020674.1	Oryzias latipes strain Hd-rR chromosome 10 sequence	84.2	250	67.6	5.22E-12
LR597463.1	Sphaeramia orbicularis genome assembly, chromosome: 6	108	244	70.082	4.57E-19
CP020639.1	Oryzias latipes strain HSOK chromosome 19	59	244	65.164	0.000208
CP020788.1	Oryzias latipes strain HNI chromosome 10	73.4	249	67.47	9.44E-09
CU929147.5	Zebrafish DNA sequence from clone CH73-194O9 in linkage group 3, complete sequence	58.1	237	65.823	0.000725
LR597476.1	Sphaeramia orbicularis genome assembly, chromosome: 19	91.5	224	69.643	3.52E-14
LR584436.1	Salmo trutta genome assembly, chromosome: 25	63.5	227	66.96	1.71E-05
LR744060.1	Scyliorhinus canicula genome assembly, chromosome: 31	64.4	222	67.568	4.89E-06
LR812496.1	Danio aesculapii genome assembly, chromosome: 17	61.7	221	70.136	5.95E-05
CP068262.1	Homo sapiens isolate CHM13 chromosome 16	63.5	202	68.317	1.71E-05
AP023476.1	Homo sapiens DNA, chromosome 16, nearly complete genome	63.5	202	68.317	1.71E-05
CP034494.1	Eukaryotic synthetic construct chromosome 16	61.7	202	67.822	5.95E-05
OE001856.1	4_Tte_b3v08	60.8	193	69.43	5.95E-05
KT991411.1	Hypsibius dujardini hypothetical protein and fushi tarazu genes, complete cds	58.1	167	68.263	0.000725
XM_034225939.1	PREDICTED: Gymnodraco acuticeps uncharacterized protein LOC793007 homolog (zgc:162331), mRNA	107	164	74.39	4.57E-19
LR633947.1	Gadus morhua genome assembly, chromosome: 5	64.4	136	71.324	4.89E-06
LR812509.1	Danio aesculapii genome assembly, chromosome: 24	59.9	304	68.75	0.000208
LR812070.1	Danio rerio genome assembly, chromosome: 8	87.8	300	68.333	4.28E-13
LR812070.1	Danio rerio genome assembly, chromosome: 8	110	295	70.169	1.31E-19
LR812070.1	Danio rerio genome assembly, chromosome: 8	97.8	272	70.221	8.27E-16
LR584426.1	Salmo trutta genome assembly, chromosome: 17	127	245	71.429	4.88E-25
LR990609.1	Phalera bucephala genome assembly, chromosome: Z	57.2	166	67.47	0.000725
LR597463.1	Sphaeramia orbicularis genome assembly, chromosome: 6	84.2	284	68.662	5.22E-12
LR584406.1	Salmo trutta genome assembly, chromosome: 6	71.6	286	67.133	3.29E-08
LR597463.1	Sphaeramia orbicularis genome assembly, chromosome: 6	96.9	281	69.395	8.27E-16
LR131971.1	Parambassis ranga genome assembly, chromosome: 20	92.4	312	67.308	3.52E-14
LR812529.1	Danio kyathit genome assembly, chromosome: 11	112	273	69.597	3.75E-20
CP020788.1	Oryzias latipes strain HNI chromosome 10	72.5	208	68.269	3.29E-08
CP009284.1	Paenibacillus sp. FSL R7-0331, complete genome	86.9	187	70.588	1.50E-12
CP020788.1	Oryzias latipes strain HNI chromosome 10	67.1	126	73.016	1.40E-06
XM_034133537.1	PREDICTED: Trematomus bernacchii uncharacterized protein LOC793007 homolog (zgc:162331), transcript variant X4, mRNA	215	274	77.372	1.23E-51
XM_034133537.1	PREDICTED: Trematomus bernacchii uncharacterized protein LOC793007 homolog (zgc:162331), transcript variant X4, mRNA	205	277	76.534	2.22E-48
XM_034133536.1	PREDICTED: Trematomus bernacchii uncharacterized protein LOC793007 homolog (zgc:162331), transcript variant X3, mRNA	215	274	77.372	1.23E-51

XM_034133536.1	PREDICTED: Trematomus bernacchii uncharacterized protein LOC793007 homolog (zgc:162331), transcript variant X3, mRNA	205	277	76.534	2.22E-48
XM_034133535.1	PREDICTED: Trematomus bernacchii uncharacterized protein LOC793007 homolog (zgc:162331), transcript variant X2, mRNA	215	274	77.372	1.23E-51
XM_034133535.1	PREDICTED: Trematomus bernacchii uncharacterized protein LOC793007 homolog (zgc:162331), transcript variant X2, mRNA	205	277	76.534	2.22E-48
XM_034133534.1	PREDICTED: Trematomus bernacchii uncharacterized protein LOC793007 homolog (zgc:162331), transcript variant X1, mRNA	215	274	77.372	1.23E-51
XM_034133534.1	PREDICTED: Trematomus bernacchii uncharacterized protein LOC793007 homolog (zgc:162331), transcript variant X1, mRNA	205	277	76.534	2.22E-48
XM_010775385.1	PREDICTED: Notothenia coriiceps mediator of RNA polymerase II transcription subunit 15-like (LOC104949106), mRNA	168	278	73.741	1.59E-37
XM_010775385.1	PREDICTED: Notothenia coriiceps mediator of RNA polymerase II transcription subunit 15-like (LOC104949106), mRNA	140	284	71.479	7.74E-29
XM_034225939.1	PREDICTED: Gymnodraco acuticeps uncharacterized protein LOC793007 homolog (zgc:162331), mRNA	188	274	75.182	1.70E-43
XM_010775385.1	PREDICTED: Notothenia coriiceps mediator of RNA polymerase II transcription subunit 15-like (LOC104949106), mRNA	200	268	76.493	9.42E-47
LR722980.1	Thalassophryne amazonica genome assembly, chromosome: 15	71.6	325	68	3.29E-08
LR722980.1	Thalassophryne amazonica genome assembly, chromosome: 15	64.4	329	67.173	4.89E-06
XM_013497760.1	Eimeria mitis uncharacterized protein (EMH_0060140), partial mRNA	67.1	285	69.825	1.40E-06
LR597458.1	Sphaeramia orbicularis genome assembly, chromosome: 1	116	283	72.438	8.83E-22
LR722980.1	Thalassophryne amazonica genome assembly, chromosome: 15	75.2	307	68.73	2.70E-09
LR722980.1	Thalassophryne amazonica genome assembly, chromosome: 15	69.8	301	68.106	1.15E-07
XM_026453704.2	PREDICTED: Piliocolobus tephrosceles probable ATP-dependent helicase PF08_0048 (LOC113224587), mRNA	119	270	70	2.53E-22
XM_026453702.2	PREDICTED: Piliocolobus tephrosceles probable ATP-dependent helicase PF08_0048 (LOC113224585), mRNA	119	270	70	2.53E-22
XM_026453701.2	PREDICTED: Piliocolobus tephrosceles serine/threonine-protein kinase fray2-like (LOC113224584), partial mRNA	119	270	70	2.53E-22
XM_026453703.1	PREDICTED: Piliocolobus tephrosceles serine/threonine-protein kinase fray2-like (LOC113224586), partial mRNA	119	270	70	2.53E-22
LR778274.1	Coregonus sp. 'balchen' genome assembly, chromosome: 22	58.1	282	67.376	0.000725
LR812532.1	Danio kyathit genome assembly, chromosome: 14	68.9	235	66.383	4.01E-07
LR812532.1	Danio kyathit genome assembly, chromosome: 14	64.4	235	65.957	4.89E-06
LR738542.1	Neostethus bicornis genome assembly, chromosome: 1	111	227	71.806	3.75E-20
XM_026453704.2	PREDICTED: Piliocolobus tephrosceles probable ATP-dependent helicase PF08_0048 (LOC113224587), mRNA	98.7	199	70.854	2.37E-16
XM_026453702.2	PREDICTED: Piliocolobus tephrosceles probable ATP-dependent helicase PF08_0048 (LOC113224585), mRNA	98.7	199	70.854	2.37E-16

XM_026453701.2	PREDICTED: Piliocolobus tephrosceles serine/threonine-protein kinase fray2-like (LOC113224584), partial mRNA	98.7	199	70.854	2.37E-16
XM_026453703.1	PREDICTED: Piliocolobus tephrosceles serine/threonine-protein kinase fray2-like (LOC113224586), partial mRNA	98.7	199	70.854	2.37E-16
OE839931.1	5_Tge_b3v08	77	203	68.966	7.74E-10
LN714486.1	TPA_asm: Neospora caninum Liverpool, chromosome chrXI, complete genome	77.9	190	68.947	7.74E-10
XM_003885574.1	Neospora caninum Liverpool conserved hypothetical protein (NCLIV_060200), partial mRNA	77.9	190	68.947	7.74E-10
FR823392.1	Neospora caninum Liverpool complete genome, chromosome XI	77.9	190	68.947	7.74E-10
LT594624.1	Plasmodium malariae genome assembly, chromosome: 3	57.2	176	67.045	0.000725
OD567462.1	4_Tbi_b3v08	63.5	171	69.591	1.71E-05
XM_016735640.1	Sporothrix schenckii 1099-18 hypothetical protein (SPSK_09059), partial mRNA	58.1	87	75.862	0.000725
LR812087.1	Danio rerio genome assembly, chromosome: 25	59.9	298	67.45	0.000208
LR597463.1	Sphaeramia orbicularis genome assembly, chromosome: 6	67.1	275	67.273	1.40E-06
LR131935.1	Cottoperca gobio genome assembly, chromosome: 5	98.7	270	68.148	2.37E-16
LR990951.1	Noctua fimbriata genome assembly, chromosome: 30	57.2	258	65.116	0.000725
LR597478.1	Sphaeramia orbicularis genome assembly, chromosome: 21	147	246	73.171	5.21E-31
LR722980.1	Thalassophryne amazonica genome assembly, chromosome: 15	90.6	310	69.677	1.23E-13
LR722980.1	Thalassophryne amazonica genome assembly, chromosome: 15	86	310	69.355	1.50E-12
LR722980.1	Thalassophryne amazonica genome assembly, chromosome: 15	84.2	311	69.453	5.22E-12
LR722980.1	Thalassophryne amazonica genome assembly, chromosome: 15	68.9	298	68.121	4.01E-07
LR812593.1	Danio rerio strain Nadia (NA) genome assembly, chromosome: 25	69.8	298	68.456	1.15E-07
LR597458.1	Sphaeramia orbicularis genome assembly, chromosome: 1	145	280	73.929	1.82E-30
LR597458.1	Sphaeramia orbicularis genome assembly, chromosome: 1	121	281	71.53	7.25E-23
LR597458.1	Sphaeramia orbicularis genome assembly, chromosome: 1	118	277	71.119	2.53E-22
XM_026453704.2	PREDICTED: Piliocolobus tephrosceles probable ATP-dependent helicase PF08_0048 (LOC113224587), mRNA	125	274	70.073	1.70E-24
XM_026453702.2	PREDICTED: Piliocolobus tephrosceles probable ATP-dependent helicase PF08_0048 (LOC113224585), mRNA	125	274	70.073	1.70E-24
XM_026453701.2	PREDICTED: Piliocolobus tephrosceles serine/threonine-protein kinase fray2-like (LOC113224584), partial mRNA	125	274	70.073	1.70E-24
XM_026453703.1	PREDICTED: Piliocolobus tephrosceles serine/threonine-protein kinase fray2-like (LOC113224586), partial mRNA	125	274	70.073	1.70E-24
LR812067.1	Danio rerio genome assembly, chromosome: 5	74.3	282	67.73	9.44E-09
LR812573.1	Danio rerio strain Nadia (NA) genome assembly, chromosome: 5	68.9	283	67.845	4.01E-07
LR812509.1	Danio aesculapii genome assembly, chromosome: 24	58.1	292	69.863	0.000725
LR597458.1	Sphaeramia orbicularis genome assembly, chromosome: 1	137	275	72.364	9.43E-28
LR597458.1	Sphaeramia orbicularis genome assembly, chromosome: 1	123	282	70.213	5.95E-24
LR597458.1	Sphaeramia orbicularis genome assembly, chromosome: 1	118	281	72.242	2.53E-22
LR812506.1	Danio aesculapii genome assembly, chromosome: 11	74.3	278	68.345	9.44E-09

LR736844.1	Pecten maximus genome assembly, chromosome: 7	72.5	315	67.302	3.29E-08
LR722980.1	Thalassophryne amazonica genome assembly, chromosome: 15	97.8	295	70.508	8.27E-16
LR722980.1	Thalassophryne amazonica genome assembly, chromosome: 15	82.4	300	69	1.82E-11
LR722980.1	Thalassophryne amazonica genome assembly, chromosome: 15	79.7	302	68.543	2.22E-10
LR722980.1	Thalassophryne amazonica genome assembly, chromosome: 15	76.1	289	69.896	2.70E-09
LR722980.1	Thalassophryne amazonica genome assembly, chromosome: 15	69.8	298	67.785	1.15E-07
LR722980.1	Thalassophryne amazonica genome assembly, chromosome: 15	67.1	287	68.293	1.40E-06
LR722980.1	Thalassophryne amazonica genome assembly, chromosome: 15	84.2	245	71.429	5.22E-12
LR722980.1	Thalassophryne amazonica genome assembly, chromosome: 15	67.1	236	70.339	1.40E-06
AP018151.1	Caenorhabditis sp. 34 TK-2017 DNA. chromosome 1, nearly complete genome	58.1	217	69.124	0.000725
LR812065.1	Danio rerio genome assembly, chromosome: 3	69.8	274	66.058	1.15E-07
XM_021472248.1	PREDICTED: Danio rerio uncharacterized LOC101885714 (LOC101885714), mRNA	69.8	274	66.058	1.15E-07
FP326669.2	Zebrafish DNA sequence from clone ZFOS-137B5 in linkage group 3, complete sequence	69.8	274	66.058	1.15E-07
LR132037.1	Anabas testudineus genome assembly, chromosome: 10	108	275	69.455	4.57E-19
LR132009.2	Betta splendens genome assembly, chromosome: 16	63.5	278	66.547	1.71E-05
LR812065.1	Danio rerio genome assembly, chromosome: 3	86	281	66.904	1.50E-12
XM_021472248.1	PREDICTED: Danio rerio uncharacterized LOC101885714 (LOC101885714), mRNA	86	281	66.904	1.50E-12
FP326669.2	Zebrafish DNA sequence from clone ZFOS-137B5 in linkage group 3, complete sequence	86	281	66.904	1.50E-12
AP022721.1	Plectropomus leopardus DNA, chromosome 22, nearly complete sequence	78.8	292	68.836	2.22E-10
LR697106.1	Chanos chanos genome assembly, chromosome: 1	64.4	285	66.316	4.89E-06
CT025742.16	Zebrafish DNA sequence from clone CH211-241D21 in linkage group 8, complete sequence	61.7	285	69.123	5.95E-05
LR597463.1	Sphaeramia orbicularis genome assembly, chromosome: 6	109	286	69.58	1.31E-19
LR597463.1	Sphaeramia orbicularis genome assembly, chromosome: 6	109	274	68.978	1.31E-19
LR597463.1	Sphaeramia orbicularis genome assembly, chromosome: 6	102	276	68.841	1.94E-17
LR584443.1	Salmo trutta genome assembly, chromosome: 40	105	280	69.643	1.60E-18
LR131935.1	Cottoperca gobio genome assembly, chromosome: 5	92.4	282	68.44	3.52E-14
LR131935.1	Cottoperca gobio genome assembly, chromosome: 5	90.6	274	67.518	1.23E-13
LR131935.1	Cottoperca gobio genome assembly, chromosome: 5	88.7	275	68	4.28E-13
LR131935.1	Cottoperca gobio genome assembly, chromosome: 5	86	274	66.788	1.50E-12
LR131935.1	Cottoperca gobio genome assembly, chromosome: 5	85.1	275	67.273	5.22E-12
LR131935.1	Cottoperca gobio genome assembly, chromosome: 5	83.3	280	66.786	1.82E-11
LR131935.1	Cottoperca gobio genome assembly, chromosome: 5	75.2	275	66.545	2.70E-09
LR131935.1	Cottoperca gobio genome assembly, chromosome: 5	73.4	289	67.128	9.44E-09
LR131935.1	Cottoperca gobio genome assembly, chromosome: 5	71.6	296	67.568	3.29E-08
LR131935.1	Cottoperca gobio genome assembly, chromosome: 5	70.7	277	66.787	1.15E-07
LR597469.1	Sphaeramia orbicularis genome assembly, chromosome: 12	79.7	290	71.034	2.22E-10
LR594556.1	Streptopelia turtur genome assembly, chromosome: 5	58.1	281	65.836	0.000725

LR738552.1	Neostethus bicornis genome assembly, chromosome: 22	76.1	284	66.549	2.70E-09
LR584436.1	Salmo trutta genome assembly, chromosome: 25	78.8	272	66.544	2.22E-10
CT025742.16	Zebrafish DNA sequence from clone CH211-241D21 in linkage group 8, complete sequence	63.5	296	69.257	1.71E-05
LR778256.1	Coregonus sp. 'balchen' genome assembly, chromosome: 4	58.1	289	65.398	0.000725
LR778256.1	Coregonus sp. 'balchen' genome assembly, chromosome: 4	61.7	284	65.493	5.95E-05
LR597467.1	Sphaeramia orbicularis genome assembly, chromosome: 10	93.3	272	67.647	1.01E-14
LR131935.1	Cottoperca gobio genome assembly, chromosome: 5	80.6	273	67.033	6.36E-11
LR131935.1	Cottoperca gobio genome assembly, chromosome: 5	77.9	271	67.897	7.74E-10
LR131935.1	Cottoperca gobio genome assembly, chromosome: 5	73.4	279	68.1	9.44E-09
LR597469.1	Sphaeramia orbicularis genome assembly, chromosome: 12	63.5	283	70.671	1.71E-05
OE019190.1	4_Tte_b3v08	72.5	275	68	3.29E-08
OD594064.1	4_Tbi_b3v08	71.6	272	68.015	3.29E-08
CP020639.1	Oryzias latipes strain HSOK chromosome 19	61.7	277	64.982	5.95E-05
AP022713.1	Plectropomus leopardus DNA, chromosome 14, nearly complete sequence	57.2	270	65.926	0.000725
LR597458.1	Sphaeramia orbicularis genome assembly, chromosome: 1	117	274	71.533	8.83E-22
LR597463.1	Sphaeramia orbicularis genome assembly, chromosome: 6	143	268	71.642	6.35E-30
LR597463.1	Sphaeramia orbicularis genome assembly, chromosome: 6	119	268	70.149	2.53E-22
LR597463.1	Sphaeramia orbicularis genome assembly, chromosome: 6	113	268	70.149	1.08E-20
LR597463.1	Sphaeramia orbicularis genome assembly, chromosome: 6	111	268	69.03	3.75E-20
LR597463.1	Sphaeramia orbicularis genome assembly, chromosome: 6	106	270	70.37	1.60E-18
LR584416.1	Salmo trutta genome assembly, chromosome: 3	145	268	72.015	1.82E-30
LR812069.1	Danio rerio genome assembly, chromosome: 7	105	279	71.685	1.60E-18
LR131935.1	Cottoperca gobio genome assembly, chromosome: 5	95.1	270	68.519	2.89E-15
LR131935.1	Cottoperca gobio genome assembly, chromosome: 5	86.9	274	67.153	1.50E-12
LR131935.1	Cottoperca gobio genome assembly, chromosome: 5	85.1	270	67.778	5.22E-12
LR131935.1	Cottoperca gobio genome assembly, chromosome: 5	82.4	282	67.73	1.82E-11
LR131935.1	Cottoperca gobio genome assembly, chromosome: 5	80.6	271	67.528	6.36E-11
LR131935.1	Cottoperca gobio genome assembly, chromosome: 5	76.1	275	66.909	2.70E-09
LR131935.1	Cottoperca gobio genome assembly, chromosome: 5	72.5	283	67.138	3.29E-08
LR131935.1	Cottoperca gobio genome assembly, chromosome: 5	69.8	270	66.296	1.15E-07
LR633949.1	Gadus morhua genome assembly, chromosome: 7	84.2	280	70	5.22E-12
OE002430.1	4_Tte_b3v08	83.3	279	68.817	1.82E-11
OE002430.1	4_Tte_b3v08	70.7	287	66.551	1.15E-07
OD570309.1	4_Tbi_b3v08	71.6	291	66.323	3.29E-08
LR597470.1	Sphaeramia orbicularis genome assembly, chromosome: 13	65.3	287	68.293	4.89E-06
LR778261.1	Coregonus sp. 'balchen' genome assembly, chromosome: 9	61.7	295	68.475	5.95E-05
LR594556.1	Streptopelia turtur genome assembly, chromosome: 5	57.2	276	65.58	0.000725
OE019190.1	4_Tte_b3v08	59	267	66.667	0.000208
OD594064.1	4_Tbi_b3v08	59	267	66.667	0.000208

LR812070.1	Danio rerio genome assembly, chromosome: 8	86.9	279	67.384	1.50E-12
LR131935.1	Cottoperca gobio genome assembly, chromosome: 5	71.6	272	66.544	3.29E-08
AP022721.1	Plectropomus leopardus DNA, chromosome 22, nearly complete sequence	72.5	276	68.478	3.29E-08
LR812496.1	Danio aesculapii genome assembly, chromosome: 17	64.4	279	68.459	4.89E-06
AC091616.2	Rattus norvegicus strain Brown Norway clone RP31-153J8, complete sequence	63.5	278	68.345	1.71E-05
AC091616.2	Rattus norvegicus strain Brown Norway clone RP31-153J8, complete sequence	61.7	272	67.279	5.95E-05
LR584441.1	Salmo trutta genome assembly, chromosome: 12	59	274	68.978	0.000208
LR597460.1	Sphaeramia orbicularis genome assembly, chromosome: 3	68	274	68.613	4.01E-07
CP020674.1	Oryzias latipes strain Hd-rR chromosome 10 sequence	75.2	270	67.037	2.70E-09
LR778277.1	Coregonus sp. 'balchen' genome assembly, chromosome: 25	66.2	277	69.314	1.40E-06
LR131935.1	Cottoperca gobio genome assembly, chromosome: 5	69.8	267	67.041	1.15E-07
LR812121.1	Erithacus rubecula genome assembly, chromosome: 18	60.8	265	69.434	5.95E-05
LR812070.1	Danio rerio genome assembly, chromosome: 8	66.2	269	69.145	1.40E-06
LR812069.1	Danio rerio genome assembly, chromosome: 7	93.3	270	70	1.01E-14
OD570309.1	4_Tbi_b3v08	71.6	266	67.669	3.29E-08
LR778261.1	Coregonus sp. 'balchen' genome assembly, chromosome: 9	65.3	286	68.881	4.89E-06
LS997562.1	Haemonchus contortus, ISE/inbred ISE, WGS project CAVP01000000 data, chromosome: 1	65.3	262	66.794	4.89E-06
LT594628.1	Plasmodium malariae genome assembly, chromosome: 7	62.6	266	65.414	1.71E-05
CP020788.1	Oryzias latipes strain HNI chromosome 10	87.8	250	68.4	4.28E-13
LR131935.1	Cottoperca gobio genome assembly, chromosome: 5	72.5	244	67.213	3.29E-08
LR584437.1	Salmo trutta genome assembly, chromosome: 21	64.4	244	66.393	4.89E-06
LR722981.1	Thalassophryne amazonica genome assembly, chromosome: 16	59	246	67.48	0.000208
CP026255.1	Scophthalmus maximus chromosome 13	76.1	265	68.679	2.70E-09
LR697110.1	Chanos chanos genome assembly, chromosome: 5	87.8	238	68.067	4.28E-13
LR584437.1	Salmo trutta genome assembly, chromosome: 21	58.1	251	66.534	0.000725
OE002430.1	4_Tte_b3v08	69.8	242	68.182	1.15E-07
LR812522.1	Danio kyathit genome assembly, chromosome: 4	78.8	240	68.75	2.22E-10
LR722980.1	Thalassophryne amazonica genome assembly, chromosome: 15	62.6	236	69.915	1.71E-05
LR597478.1	Sphaeramia orbicularis genome assembly, chromosome: 21	89.7	194	70.103	1.23E-13
LR812517.1	Danio aesculapii genome assembly, chromosome: 3	57.2	201	68.657	0.000725
LR597463.1	Sphaeramia orbicularis genome assembly, chromosome: 6	92.4	175	71.429	3.52E-14
OE002430.1	4_Tte_b3v08	70.7	182	70.879	1.15E-07
CT025742.16	Zebrafish DNA sequence from clone CH211-241D21 in linkage group 8, complete sequence	59	191	71.204	0.000208
LR744060.1	Scyliorhinus canicula genome assembly, chromosome: 31	67.1	151	69.536	1.40E-06
LR584407.1	Salmo trutta genome assembly, chromosome: 8	61.7	156	70.513	5.95E-05
LR738551.1	Neostethus bicornis genome assembly, chromosome: 11	65.3	154	70.779	4.89E-06
OD570309.1	4_Tbi_b3v08	60.8	146	69.863	5.95E-05
XM_038360266.1	PREDICTED: Zerene cesonia activated Cdc42 kinase-like (LOC119835447), mRNA	78.8	113	75.221	2.22E-10

XM_009033279.1	Helobdella robusta hypothetical protein partial mRNA	60.8	98	73.469	5.95E-05
CP027285.1	Lateolabrax maculatus chromosome Lm24	75.2	86	79.07	2.70E-09
LR736843.1	Pecten maximus genome assembly, chromosome: 6	57.2	72	80.556	0.000725
LR131935.1	Cottoperca gobio genome assembly, chromosome: 5	83.3	276	67.391	1.82E-11
LR131935.1	Cottoperca gobio genome assembly, chromosome: 5	85.1	293	67.577	5.22E-12
LR131935.1	Cottoperca gobio genome assembly, chromosome: 5	64.4	276	66.304	4.89E-06
LR812525.1	Danio kyathit genome assembly, chromosome: 7	63.5	274	67.153	1.71E-05
XM_010775385.1	PREDICTED: Notothenia coriiceps mediator of RNA polymerase II transcription subunit 15-like (LOC104949106), mRNA	86	287	67.944	1.50E-12
LR584432.1	Salmo trutta genome assembly, chromosome: 30	137	267	71.536	9.43E-28
LR131935.1	Cottoperca gobio genome assembly, chromosome: 5	69.8	268	66.791	1.15E-07
LR697110.1	Chanos chanos genome assembly, chromosome: 5	90.6	257	67.704	1.23E-13
LR697110.1	Chanos chanos genome assembly, chromosome: 5	72.5	257	66.148	3.29E-08
LR812040.1	Danio rerio genome assembly, chromosome: 3	69.8	273	69.231	1.15E-07
LR744056.1	Scyliorhinus canicula genome assembly, chromosome: 27	59.9	255	67.843	0.000208
LR584432.1	Salmo trutta genome assembly, chromosome: 30	102	221	71.493	1.94E-17
LS423616.1	Anas platyrhynchos genome assembly, chromosome: 6	59.9	161	71.429	0.000208
CP002659.1	Sphaerochaeta coccoides DSM 17374, complete genome	70.7	76	80.263	1.15E-07
LR812554.1	Danio rerio strain Cooch Behar (CB) genome assembly, chromosome: 11	64.4	287	68.641	4.89E-06
LR131928.1	Cottoperca gobio genome assembly, chromosome: 20	138	266	71.429	2.70E-28
LR812073.1	Danio rerio genome assembly, chromosome: 11	99.6	293	71.672	2.37E-16
LR633945.1	Gadus morhua genome assembly, chromosome: 3	71.6	188	70.745	3.29E-08
LR880663.1	Poecilia reticulata genome assembly, chromosome: 19	71.6	283	67.845	3.29E-08
CP020639.1	Oryzias latipes strain HSOK chromosome 19	65.3	277	65.343	4.89E-06
LR812045.1	Danio rerio genome assembly, chromosome: 8	68	279	67.742	4.01E-07
LR812079.1	Danio rerio genome assembly, chromosome: 17	57.2	283	67.138	0.000725
CP068262.1	Homo sapiens isolate CHM13 chromosome 16	82.4	275	67.636	1.82E-11
AP023476.1	Homo sapiens DNA, chromosome 16, nearly complete genome	82.4	275	67.636	1.82E-11
CP034494.1	Eukaryotic synthetic construct chromosome 16	73.4	265	67.17	9.44E-09
LR812045.1	Danio rerio genome assembly, chromosome: 8	83.3	283	68.551	1.82E-11
LR812045.1	Danio rerio genome assembly, chromosome: 8	69.8	292	67.123	1.15E-07
LR597475.1	Sphaeramia orbicularis genome assembly, chromosome: 18	62.6	270	67.407	1.71E-05
LR584406.1	Salmo trutta genome assembly, chromosome: 6	73.4	270	66.667	9.44E-09
LR722978.1	Thalassophryne amazonica genome assembly, chromosome: 13	58.1	263	66.16	0.000725
LR584443.1	Salmo trutta genome assembly, chromosome: 40	96.9	244	70.082	8.27E-16
AC092341.5	Homo sapiens chromosome 16 clone RP11-260K13, complete sequence	69.8	238	68.067	1.15E-07
XM_027383799.1	PREDICTED: Penaeus vannamei trichohyalin-like (LOC113830593), mRNA	67.1	222	68.919	1.40E-06
LR584398.1	Rhinatrema bivittatum genome assembly, chromosome: 12	59	205	69.756	0.000208
LR131992.1	Gouania willdenowi genome assembly, chromosome: 16	65.3	147	70.748	4.89E-06
LR633947.1	Gadus morhua genome assembly, chromosome: 5	68	82	78.049	4.01E-07

LR131928.1	Cottoperca gobio genome assembly, chromosome: 20	135	266	71.805	3.29E-27
LR584045.1	Echeneis naucrates genome assembly, chromosome: 4	61.7	236	67.797	5.95E-05
LR812529.1	Danio kyathit genome assembly, chromosome: 11	78.8	283	66.784	2.22E-10
LR812544.1	Danio rerio strain Cooch Behar (CB) genome assembly, chromosome: 1	74.3	113	74.336	9.44E-09
OE000218.1	4_Tte_b3v08	88.7	302	66.556	4.28E-13
LR990951.1	Noctua fimbriata genome assembly, chromosome: 30	61.7	268	65.299	5.95E-05
LR584445.1	Salmo trutta genome assembly, chromosome: 2	163	266	75.94	6.78E-36
OE839931.1	5_Tge_b3v08	86	267	67.79	1.50E-12
CR385022.10	Zebrafish DNA sequence from clone CH211-63C12 in linkage group 23, complete sequence	80.6	265	72.453	6.36E-11
LR633949.1	Gadus morhua genome assembly, chromosome: 7	82.4	269	69.517	1.82E-11
LR812083.1	Danio rerio genome assembly, chromosome: 21	58.1	259	69.884	0.000725
LR990951.1	Noctua fimbriata genome assembly, chromosome: 30	63.5	247	65.587	1.71E-05
LR812540.1	Danio kyathit genome assembly, chromosome: 22	58.1	262	69.466	0.000725
LR812540.1	Danio kyathit genome assembly, chromosome: 22	60.8	215	70.233	5.95E-05
LN714486.1	TPA_asm: Neospora caninum Liverpool, chromosome chrXI, complete genome	70.7	196	67.857	1.15E-07
XM_003885574.1	Neospora caninum Liverpool conserved hypothetical protein (NCLIV_060200), partial mRNA	70.7	196	67.857	1.15E-07
FR823392.1	Neospora caninum Liverpool complete genome, chromosome XI	70.7	196	67.857	1.15E-07
LT594624.1	Plasmodium malariae genome assembly, chromosome: 3	63.5	191	67.539	1.71E-05
OE000218.1	4_Tte_b3v08	72.5	191	69.634	3.29E-08
OE839931.1	5_Tge_b3v08	59.9	99	73.737	0.000208
LR129761.1	Plasmodium yoelii killicki strain 193L genome assembly, chromosome: 6	65.3	93	75.269	4.89E-06
LR738542.1	Neostethus bicornis genome assembly, chromosome: 1	87.8	271	67.528	4.28E-13
LR812529.1	Danio kyathit genome assembly, chromosome: 11	116	267	71.161	8.83E-22
LR778284.1	Coregonus sp. 'balchen' genome assembly, chromosome: 32	65.3	277	71.119	4.89E-06
LR778274.1	Coregonus sp. 'balchen' genome assembly, chromosome: 22	69.8	261	68.582	1.15E-07
LR990951.1	Noctua fimbriata genome assembly, chromosome: 30	67.1	246	66.26	1.40E-06
LR990951.1	Noctua fimbriata genome assembly, chromosome: 30	61.7	246	65.447	5.95E-05
LR812065.1	Danio rerio genome assembly, chromosome: 3	61.7	196	67.857	5.95E-05
LR812040.1	Danio rerio genome assembly, chromosome: 3	57.2	196	67.347	0.000725
FP236320.5	Zebrafish DNA sequence from clone CH1073-504A6 in linkage group 3, complete sequence	57.2	196	67.347	0.000725
FP326653.4	Zebrafish DNA sequence from clone CH1073-480I21 in linkage group 3, complete sequence	57.2	196	67.347	0.000725
LR722980.1	Thalassophryne amazonica genome assembly, chromosome: 15	73.4	307	68.078	9.44E-09
LR594556.1	Streptopelia turtur genome assembly, chromosome: 5	65.3	268	66.418	4.89E-06
LR597463.1	Sphaeramia orbicularis genome assembly, chromosome: 6	66.2	264	67.803	1.40E-06
LR597463.1	Sphaeramia orbicularis genome assembly, chromosome: 6	93.3	263	68.821	1.01E-14
LR597472.1	Sphaeramia orbicularis genome assembly, chromosome: 15	77.9	259	67.568	7.74E-10

OE840970.1	5_Tge_b3v08	63.5	274	67.883	1.71E-05
LR722980.1	Thalassophryne amazonica genome assembly, chromosome: 15	77.9	304	68.75	7.74E-10
LR722980.1	Thalassophryne amazonica genome assembly, chromosome: 15	77	277	68.953	7.74E-10
LR722980.1	Thalassophryne amazonica genome assembly, chromosome: 15	72.5	277	68.592	3.29E-08
LR722980.1	Thalassophryne amazonica genome assembly, chromosome: 15	68.9	300	67.667	4.01E-07
LR722980.1	Thalassophryne amazonica genome assembly, chromosome: 15	60.8	297	67.003	5.95E-05
LR597472.1	Sphaeramia orbicularis genome assembly, chromosome: 15	75.2	257	68.093	2.70E-09
LS997562.1	Haemonchus contortus, ISE/inbred ISE, WGS project CAVP01000000 data, chromosome: 1	57.2	254	68.504	0.000725
LR597458.1	Sphaeramia orbicularis genome assembly, chromosome: 1	71.6	281	67.972	3.29E-08
LR812496.1	Danio aesculapii genome assembly, chromosome: 17	59	231	71.429	0.000208
LR722980.1	Thalassophryne amazonica genome assembly, chromosome: 15	68	239	69.874	4.01E-07
XM_023483289.1	PREDICTED: Eurytemora affinis uncharacterized LOC111709561 (LOC111709561), mRNA	72.5	110	80.909	3.29E-08
LR597476.1	Sphaeramia orbicularis genome assembly, chromosome: 19	105	256	70.312	1.60E-18
LR778258.1	Coregonus sp. 'balchen' genome assembly, chromosome: 6	68	217	68.203	4.01E-07
LR761660.1	Aphantopus hyperantus genome assembly, chromosome: 13	59.9	148	69.595	0.000208
XM_009033279.1	Helobdella robusta hypothetical protein partial mRNA	58.1	124	70.161	0.000725
OE843916.1	5_Tge_b3v08	59.9	260	65	0.000208
LR131935.1	Cottoperca gobio genome assembly, chromosome: 5	81.5	265	67.547	6.36E-11
LR812506.1	Danio aesculapii genome assembly, chromosome: 11	71.6	265	67.547	3.29E-08
OD570309.1	4_Tbi_b3v08	83.3	265	69.434	1.82E-11
LR812522.1	Danio kyathit genome assembly, chromosome: 4	71.6	272	68.015	3.29E-08
LR131935.1	Cottoperca gobio genome assembly, chromosome: 5	72.5	259	66.409	3.29E-08
LR597465.1	Sphaeramia orbicularis genome assembly, chromosome: 8	60.8	258	65.891	5.95E-05
CT025742.16	Zebrafish DNA sequence from clone CH211-241D21 in linkage group 8, complete sequence	70.7	268	69.776	1.15E-07
CP020674.1	Oryzias latipes strain Hd-rR chromosome 10 sequence	72.5	243	67.078	3.29E-08
LR584437.1	Salmo trutta genome assembly, chromosome: 21	68.9	234	67.949	4.01E-07
AC092341.5	Homo sapiens chromosome 16 clone RP11-260K13, complete sequence	59	218	66.514	0.000208
CP020674.1	Oryzias latipes strain Hd-rR chromosome 10 sequence	79.7	205	68.78	2.22E-10
LR990668.1	Xestia xanthographa genome assembly, chromosome: 27	59.9	200	66.5	0.000208
LR812070.1	Danio rerio genome assembly, chromosome: 8	68.9	202	72.277	4.01E-07
CT025742.16	Zebrafish DNA sequence from clone CH211-241D21 in linkage group 8, complete sequence	58.1	206	69.903	0.000725
LR584412.1	Salmo trutta genome assembly, chromosome: 24	59	196	70.918	0.000208
LR812511.1	Danio aesculapii genome assembly, chromosome: 2	57.2	195	67.692	0.000725
LR131916.1	Cottoperca gobio genome assembly, chromosome: 1	87.8	157	75.796	4.28E-13
LR597479.1	Sphaeramia orbicularis genome assembly, chromosome: 22	59.9	152	69.079	0.000208
LR990260.1	Aricia agestis genome assembly, chromosome: 4	57.2	91	73.626	0.000725

CP032597.1	Lateolabrax maculatus linkage group 22 sequence	59.9	90	74.444	0.000208
LR131935.1	Cottoperca gobio genome assembly, chromosome: 5	72.5	256	66.406	3.29E-08
LR131935.1	Cottoperca gobio genome assembly, chromosome: 5	61.7	250	66	5.95E-05
XM_027158426.1	PREDICTED: Tachysurus fulvidraco splicing factor U2af large subunit A-like (LOC113650229), mRNA	83.3	250	67.6	1.82E-11
LR899861.1	Darwinula stevensoni	70.7	141	70.922	1.15E-07
CP027285.1	Lateolabrax maculatus chromosome Lm24	67.1	89	76.404	1.40E-06
CP032597.1	Lateolabrax maculatus linkage group 22 sequence	67.1	74	79.73	1.40E-06
LR736851.1	Pecten maximus genome assembly, chromosome: 14	69.8	68	82.353	1.15E-07
LR812577.1	Danio rerio strain Nadia (NA) genome assembly, chromosome: 9	59	259	68.726	0.000208
LR697110.1	Chanos chanos genome assembly, chromosome: 5	76.1	229	67.249	2.70E-09
LR812506.1	Danio aesculapii genome assembly, chromosome: 11	59	230	67.826	0.000208
LR778269.1	Coregonus sp. 'balchen' genome assembly, chromosome: 17	60.8	170	68.824	5.95E-05
XM_027383799.1	PREDICTED: Penaeus vannamei trichohyalin-like (LOC113830593), mRNA	59.9	226	69.912	0.000208
CP009284.1	Paenibacillus sp. FSL R7-0331, complete genome	78.8	202	69.307	2.22E-10
LR597467.1	Sphaeramia orbicularis genome assembly, chromosome: 10	93.3	287	67.247	1.01E-14
LR131928.1	Cottoperca gobio genome assembly, chromosome: 20	136	254	72.441	9.43E-28
CP004006.1	Legionella oakridgensis ATCC 33761 = DSM 21215, complete genome	57.2	81	75.309	0.000725
LN697154.1	Cucumis melo genomic scaffold, unassembled_sequence45415	57.2	66	78.788	0.000725
LR722980.1	Thalassophryne amazonica genome assembly, chromosome: 15	76.1	275	67.636	2.70E-09
CR385022.10	Zebrafish DNA sequence from clone CH211-63C12 in linkage group 23, complete sequence	83.3	234	73.504	1.82E-11
LN714486.1	TPA_asm: Neospora caninum Liverpool, chromosome chrXI, complete genome	65.3	203	66.995	4.89E-06
XM_003885574.1	Neospora caninum Liverpool conserved hypothetical protein (NCLIV_060200), partial mRNA	65.3	203	66.995	4.89E-06
FR823392.1	Neospora caninum Liverpool complete genome, chromosome XI	65.3	203	66.995	4.89E-06
LR990268.1	Aricia agestis genome assembly, chromosome: 12	57.2	85	75.294	0.000725
OC475855.1	3_Tce_b3v08	61.7	220	68.636	5.95E-05
LR812512.1	Danio aesculapii genome assembly, chromosome: 7	57.2	205	71.22	0.000725
LR594556.1	Streptopelia turtur genome assembly, chromosome: 5	62.6	250	66.4	1.71E-05
CR385022.10	Zebrafish DNA sequence from clone CH211-63C12 in linkage group 23, complete sequence	77	247	72.065	7.74E-10
XM_001524819.1	Lodderomyces elongisporus NRRL YB-4239 conserved hypothetical protein (LELG_03901) partial mRNA	67.1	175	68.571	1.40E-06
LS992271.1	Candida hispaniensis genome assembly, chromosome: B	59.9	132	75	0.000208
LR899861.1	Darwinula stevensoni	67.1	129	71.318	1.40E-06
CP002659.1	Sphaerochaeta coccoides DSM 17374, complete genome	59	91	75.824	0.000208
LR131999.1	Gouania willdenowi genome assembly, chromosome: 13	108	238	70.588	4.57E-19
CP032605.1	Lateolabrax maculatus linkage group 8 sequence	60.8	75	78.667	5.95E-05
CP027276.1	Lateolabrax maculatus chromosome Lm15	60.8	75	78.667	5.95E-05

LR812501.1	Danio aesculapii genome assembly, chromosome: 21	57.2	263	68.821	0.000725
LR812124.1	Erithacus rubecula genome assembly, chromosome: 21	59	259	66.795	0.000208
LR597458.1	Sphaeramia orbicularis genome assembly, chromosome: 1	128	253	73.518	4.88E-25
LR597464.1	Sphaeramia orbicularis genome assembly, chromosome: 7	114	258	70.93	3.08E-21
LR812538.1	Danio kyathit genome assembly, chromosome: 20	81.5	266	71.805	6.36E-11
LR633945.1	Gadus morhua genome assembly, chromosome: 3	75.2	198	70.202	2.70E-09
LR812522.1	Danio kyathit genome assembly, chromosome: 4	59	191	68.586	0.000208
LR812070.1	Danio rerio genome assembly, chromosome: 8	69.8	190	72.632	1.15E-07
LR738551.1	Neostethus bicornis genome assembly, chromosome: 11	65.3	162	70.988	4.89E-06
LR131935.1	Cottoperca gobio genome assembly, chromosome: 5	66.2	243	66.667	1.40E-06
LR697110.1	Chanos chanos genome assembly, chromosome: 5	78.8	233	67.382	2.22E-10
OD662508.1	4_Tbi_b3v08	57.2	250	68	0.000725
OA566717.1	1_Tdi_b3v08	63.5	203	69.458	1.71E-05
LR597457.1	Salarias fasciatus genome assembly, chromosome: 23	58.1	207	70.531	0.000725
CP062022.1	Macrobrachium nipponense isolate FS-2020 chromosome 18	59.9	204	70.098	0.000208
XM_024887284.1	Hyaloscypha bicolor E uncharacterized protein (K444DRAFT_667332), mRNA	58.1	59	81.356	0.000725
LR778266.1	Coregonus sp. 'balchen' genome assembly, chromosome: 14	68	178	67.416	4.01E-07
LR633961.1	Gadus morhua genome assembly, chromosome: 19	62.6	253	68.775	1.71E-05
AP022720.1	Plectropomus leopardus DNA, chromosome 21, nearly complete sequence	57.2	51	84.314	0.000725
CP004006.1	Legionella oakridgensis ATCC 33761 = DSM 21215, complete genome	61.7	81	76.543	5.95E-05
LN714486.1	TPA_asm: Neospora caninum Liverpool, chromosome chrXI, complete genome	69.8	203	67.488	1.15E-07
XM_003885574.1	Neospora caninum Liverpool conserved hypothetical protein (NCLIV_060200), partial mRNA	69.8	203	67.488	1.15E-07
FR823392.1	Neospora caninum Liverpool complete genome, chromosome XI	69.8	203	67.488	1.15E-07
LT594624.1	Plasmodium malariae genome assembly, chromosome: 3	61.7	176	67.614	5.95E-05
LR722980.1	Thalassophryne amazonica genome assembly, chromosome: 15	76.1	268	69.403	2.70E-09
LR722980.1	Thalassophryne amazonica genome assembly, chromosome: 15	73.4	271	70.111	9.44E-09
LR722980.1	Thalassophryne amazonica genome assembly, chromosome: 15	60.8	294	67.687	5.95E-05
LR584417.1	Salmo trutta genome assembly, chromosome: 37	96.9	256	71.875	8.27E-16
LR594556.1	Streptopelia turtur genome assembly, chromosome: 5	62.6	252	66.667	1.71E-05
LR722980.1	Thalassophryne amazonica genome assembly, chromosome: 15	77	263	69.962	7.74E-10
LR722980.1	Thalassophryne amazonica genome assembly, chromosome: 15	75.2	263	68.061	2.70E-09
LR722980.1	Thalassophryne amazonica genome assembly, chromosome: 15	73.4	267	70.037	9.44E-09
LR722980.1	Thalassophryne amazonica genome assembly, chromosome: 15	69.8	261	69.732	1.15E-07
LR722980.1	Thalassophryne amazonica genome assembly, chromosome: 15	66.2	265	67.925	1.40E-06
LR722980.1	Thalassophryne amazonica genome assembly, chromosome: 15	60.8	260	68.077	5.95E-05
OE002430.1	4_Tte_b3v08	77.9	243	68.724	7.74E-10
LR722980.1	Thalassophryne amazonica genome assembly, chromosome: 15	77.9	272	69.485	7.74E-10
LR722980.1	Thalassophryne amazonica genome assembly, chromosome: 15	75.2	263	68.821	2.70E-09
LR722980.1	Thalassophryne amazonica genome assembly, chromosome: 15	70.7	263	68.441	1.15E-07

LR722980.1	Thalassophryne amazonica genome assembly, chromosome: 15	70.7	246	69.919	1.15E-07
LR722980.1	Thalassophryne amazonica genome assembly, chromosome: 15	66.2	259	69.112	1.40E-06
LR722980.1	Thalassophryne amazonica genome assembly, chromosome: 15	63.5	270	67.778	1.71E-05
LR584417.1	Salmo trutta genome assembly, chromosome: 37	104	234	73.077	5.57E-18
LR812042.1	Danio rerio genome assembly, chromosome: 5	57.2	227	68.282	0.000725
LR722980.1	Thalassophryne amazonica genome assembly, chromosome: 15	63.5	235	67.234	1.71E-05
LR722980.1	Thalassophryne amazonica genome assembly, chromosome: 15	61.7	211	68.246	5.95E-05
LR722980.1	Thalassophryne amazonica genome assembly, chromosome: 15	63.5	204	69.608	1.71E-05
OE000218.1	4_Tte_b3v08	61.7	261	65.9	5.95E-05
LR597464.1	Sphaeramia orbicularis genome assembly, chromosome: 7	102	244	70.492	1.94E-17
LR535816.1	Denticeps clupeoides genome assembly, chromosome: 4	57.2	233	66.524	0.000725
OE839931.1	5_Tge_b3v08	77	246	68.293	7.74E-10
OD567462.1	4_Tbi_b3v08	89.7	240	68.75	1.23E-13
LR697116.1	Chanos chanos genome assembly, chromosome: 11	85.1	235	68.936	5.22E-12
CP026255.1	Scophthalmus maximus chromosome 13	72.5	248	69.355	3.29E-08
LR990653.1	Xestia xanthographa genome assembly, chromosome: 12	75.2	212	68.396	2.70E-09
LR744060.1	Scyliorhinus canicula genome assembly, chromosome: 31	68	187	68.449	4.01E-07
LR697116.1	Chanos chanos genome assembly, chromosome: 11	57.2	163	70.552	0.000725
XM_038360266.1	PREDICTED: Zerene cesonia activated Cdc42 kinase-like (LOC119835447), mRNA	65.3	113	72.566	4.89E-06
CP027285.1	Lateolabrax maculatus chromosome Lm24	66.2	86	76.744	1.40E-06
CP032600.1	Lateolabrax maculatus linkage group 3 sequence	60.8	78	76.923	5.95E-05
LR584406.1	Salmo trutta genome assembly, chromosome: 6	59	248	67.339	0.000208
LR584417.1	Salmo trutta genome assembly, chromosome: 37	81.5	237	70.464	6.36E-11
LR584416.1	Salmo trutta genome assembly, chromosome: 3	88.7	248	71.371	4.28E-13
LR584416.1	Salmo trutta genome assembly, chromosome: 3	74.3	239	70.293	9.44E-09
LR584416.1	Salmo trutta genome assembly, chromosome: 3	74.3	239	71.13	9.44E-09
LR584417.1	Salmo trutta genome assembly, chromosome: 37	99.6	237	72.152	2.37E-16
LR584417.1	Salmo trutta genome assembly, chromosome: 37	97.8	234	73.077	8.27E-16
LR584417.1	Salmo trutta genome assembly, chromosome: 37	95.1	242	71.488	2.89E-15
LR584417.1	Salmo trutta genome assembly, chromosome: 37	91.5	234	71.795	3.52E-14
LR584417.1	Salmo trutta genome assembly, chromosome: 37	81.5	231	70.996	6.36E-11
LR584417.1	Salmo trutta genome assembly, chromosome: 37	73.4	240	70.417	9.44E-09
LR584417.1	Salmo trutta genome assembly, chromosome: 37	94.2	230	72.174	1.01E-14
LR597460.1	Sphaeramia orbicularis genome assembly, chromosome: 3	69.8	207	67.633	1.15E-07
LR812584.1	Danio rerio strain Nadia (NA) genome assembly, chromosome: 16	69.8	213	71.362	1.15E-07
LR584416.1	Salmo trutta genome assembly, chromosome: 3	80.6	184	72.826	6.36E-11
LR584416.1	Salmo trutta genome assembly, chromosome: 3	63.5	179	71.508	1.71E-05
LR584417.1	Salmo trutta genome assembly, chromosome: 37	65.3	190	69.474	4.89E-06
LR584417.1	Salmo trutta genome assembly, chromosome: 37	91.5	174	75.287	3.52E-14

LR584417.1	Salmo trutta genome assembly, chromosome: 37	63.5	176	71.591	1.71E-05
LR584417.1	Salmo trutta genome assembly, chromosome: 37	57.2	104	75	0.000725
XM_001527872.1	Lodderomyces elongisporus NRRL YB-4239 hypothetical protein (LELG_00442) partial mRNA	63.5	87	75.862	1.71E-05
XM_027383799.1	PREDICTED: Penaeus vannamei trichohyalin-like (LOC113830593), mRNA	61.7	239	70.293	5.95E-05
AP022720.1	Plectropomus leopardus DNA, chromosome 21, nearly complete sequence	57.2	46	86.957	0.000725
XM_007683842.1	Baudoinia panamericana UAMH 10762 uncharacterized protein (BAUCODRAFT_152863), partial mRNA	59	237	67.089	0.000208
LR131928.1	Cottoperca gobio genome assembly, chromosome: 20	57.2	220	68.636	0.000725
LR131928.1	Cottoperca gobio genome assembly, chromosome: 20	57.2	220	67.727	0.000725
CP004006.1	Legionella oakridgensis ATCC 33761 = DSM 21215, complete genome	59	77	76.623	0.000208
LR990951.1	Noctua fimbriata genome assembly, chromosome: 30	69.8	223	66.816	1.15E-07
LR990951.1	Noctua fimbriata genome assembly, chromosome: 30	66.2	223	66.816	1.40E-06
LR990951.1	Noctua fimbriata genome assembly, chromosome: 30	70.7	211	67.299	1.15E-07
LT594624.1	Plasmodium malariae genome assembly, chromosome: 3	63.5	172	68.023	1.71E-05
LR990951.1	Noctua fimbriata genome assembly, chromosome: 30	70.7	231	66.667	1.15E-07
LR990951.1	Noctua fimbriata genome assembly, chromosome: 30	67.1	231	66.667	1.40E-06
LR990951.1	Noctua fimbriata genome assembly, chromosome: 30	60.8	230	66.087	5.95E-05
XM_013497760.1	Eimeria mitis uncharacterized protein (EMH_0060140), partial mRNA	65.3	230	70.435	4.89E-06
LR738542.1	Neostethus bicornis genome assembly, chromosome: 1	94.2	224	69.196	1.01E-14
LR990951.1	Noctua fimbriata genome assembly, chromosome: 30	68	222	66.667	4.01E-07
LR990951.1	Noctua fimbriata genome assembly, chromosome: 30	63.5	222	66.216	1.71E-05
LR990951.1	Noctua fimbriata genome assembly, chromosome: 30	59	222	65.766	0.000208
LR584416.1	Salmo trutta genome assembly, chromosome: 3	79.7	226	71.681	2.22E-10
LR990951.1	Noctua fimbriata genome assembly, chromosome: 30	59.9	210	66.19	0.000208
LR812554.1	Danio rerio strain Cooch Behar (CB) genome assembly, chromosome: 11	68.9	191	69.634	4.01E-07
LR812070.1	Danio rerio genome assembly, chromosome: 8	71.6	233	71.245	3.29E-08
LR812070.1	Danio rerio genome assembly, chromosome: 8	57.2	230	69.565	0.000725
LR736840.1	Pecten maximus genome assembly, chromosome: 3	72.5	243	70.37	3.29E-08
LR812070.1	Danio rerio genome assembly, chromosome: 8	66.2	234	69.658	1.40E-06
LR812506.1	Danio aesculapii genome assembly, chromosome: 11	77	236	69.068	7.74E-10
LR812040.1	Danio rerio genome assembly, chromosome: 3	72.5	233	70.386	3.29E-08
CT025742.16	Zebrafish DNA sequence from clone CH211-241D21 in linkage group 8, complete sequence	71.6	231	70.563	3.29E-08
LR584417.1	Salmo trutta genome assembly, chromosome: 37	81.5	232	71.983	6.36E-11
LR584417.1	Salmo trutta genome assembly, chromosome: 37	75.2	230	70.435	2.70E-09
LR584417.1	Salmo trutta genome assembly, chromosome: 37	61.7	238	68.908	5.95E-05
OE839931.1	5_Tge_b3v08	103	217	70.968	1.94E-17
LR812538.1	Danio kyathit genome assembly, chromosome: 20	68.9	225	69.333	4.01E-07
LR812540.1	Danio kyathit genome assembly, chromosome: 22	57.2	224	68.304	0.000725

LR584416.1	Salmo trutta genome assembly, chromosome: 3	84.2	229	71.616	5.22E-12
LR812070.1	Danio rerio genome assembly, chromosome: 8	62.6	216	70.833	1.71E-05
CT025742.16	Zebrafish DNA sequence from clone CH211-241D21 in linkage group 8, complete sequence	70.7	216	71.296	1.15E-07
LR812506.1	Danio aesculapii genome assembly, chromosome: 11	68.9	210	68.095	4.01E-07
LR584417.1	Salmo trutta genome assembly, chromosome: 37	74.3	173	72.254	9.44E-09
OD567462.1	4_Tbi_b3v08	78.8	165	70.909	2.22E-10
OE000218.1	4_Tte_b3v08	83.3	165	71.515	1.82E-11
LR584416.1	Salmo trutta genome assembly, chromosome: 3	62.6	133	73.684	1.71E-05
LR990653.1	Xestia xanthographa genome assembly, chromosome: 12	59.9	129	70.543	0.000208
CP032600.1	Lateolabrax maculatus linkage group 3 sequence	63.5	110	73.636	1.71E-05
OE000218.1	4_Tte_b3v08	85.1	176	71.023	5.22E-12
CP009515.1	Methanosarcina lacustris Z-7289, complete genome	60.8	101	78.218	5.95E-05
LR990951.1	Noctua fimbriata genome assembly, chromosome: 30	65.3	228	66.228	4.89E-06
OE001856.1	4_Tte_b3v08	67.1	215	68.372	1.40E-06
AL928589.11	Mouse DNA sequence from clone RP23-350C1 on chromosome 2, complete sequence	59	203	66.502	0.000208
CR352330.14	Mouse DNA sequence from clone RP23-374F1 on chromosome X, complete sequence	57.2	167	70.659	0.000725
LR812045.1	Danio rerio genome assembly, chromosome: 8	63.5	126	74.603	1.71E-05
LR812576.1	Danio rerio strain Nadia (NA) genome assembly, chromosome: 8	63.5	126	74.603	1.71E-05
LR584417.1	Salmo trutta genome assembly, chromosome: 37	96	226	72.566	2.89E-15
LT594624.1	Plasmodium malariae genome assembly, chromosome: 3	71.6	159	69.811	3.29E-08
LR736844.1	Pecten maximus genome assembly, chromosome: 7	64.4	241	68.465	4.89E-06
LR736844.1	Pecten maximus genome assembly, chromosome: 7	64.4	253	67.984	4.89E-06
LR990268.1	Aricia agestis genome assembly, chromosome: 12	74.3	217	67.742	9.44E-09
LR597458.1	Sphaeramia orbicularis genome assembly, chromosome: 1	62.6	208	66.827	1.71E-05
OE839931.1	5_Tge_b3v08	73.4	241	67.635	9.44E-09
LR722978.1	Thalassophryne amazonica genome assembly, chromosome: 13	59	163	68.098	0.000208
LR129761.1	Plasmodium yoelii killicki strain 193L genome assembly, chromosome: 6	69.8	115	73.913	1.15E-07
LN714486.1	TPA_asm: Neospora caninum Liverpool, chromosome chrXI, complete genome	68	197	67.513	4.01E-07
XM_003885574.1	Neospora caninum Liverpool conserved hypothetical protein (NCLIV_060200), partial mRNA	68	197	67.513	4.01E-07
FR823392.1	Neospora caninum Liverpool complete genome, chromosome XI	68	197	67.513	4.01E-07
LN714486.1	TPA_asm: Neospora caninum Liverpool, chromosome chrXI, complete genome	74.3	178	69.101	9.44E-09
XM_003885574.1	Neospora caninum Liverpool conserved hypothetical protein (NCLIV_060200), partial mRNA	74.3	178	69.101	9.44E-09
FR823392.1	Neospora caninum Liverpool complete genome, chromosome XI	74.3	178	69.101	9.44E-09
OB787669.1	Cyprideis torosa	68.9	141	74.468	4.01E-07
LR812544.1	Danio rerio strain Cooch Behar (CB) genome assembly, chromosome: 1	62.6	99	73.737	1.71E-05
CP004006.1	Legionella oakridgensis ATCC 33761 = DSM 21215, complete genome	58.1	79	75.949	0.000725
LR597464.1	Sphaeramia orbicularis genome assembly, chromosome: 7	115	214	72.897	3.08E-21

LR812512.1	Danio aesculapii genome assembly, chromosome: 7	59	206	70.874	0.000208
LR812593.1	Danio rerio strain Nadia (NA) genome assembly, chromosome: 25	67.1	214	70.093	1.40E-06
CP009284.1	Paenibacillus sp. FSL R7-0331, complete genome	77	187	68.984	7.74E-10
LR597477.1	Sphaeramia orbicularis genome assembly, chromosome: 20	69.8	83	78.313	1.15E-07
OD570309.1	4_Tbi_b3v08	64.4	223	69.058	4.89E-06
LR722980.1	Thalassophryne amazonica genome assembly, chromosome: 15	65.3	215	68.372	4.89E-06
LR722980.1	Thalassophryne amazonica genome assembly, chromosome: 15	59.9	199	68.342	0.000208
XM_022967385.1	PREDICTED: Spodoptera litura U1 small nuclear ribonucleoprotein 70 kDa-like (LOC111354103), transcript variant X2, mRNA	59.9	115	71.304	0.000208
XM_022967384.1	PREDICTED: Spodoptera litura U1 small nuclear ribonucleoprotein 70 kDa-like (LOC111354103), transcript variant X1, mRNA	59.9	115	71.304	0.000208
LR736851.1	Pecten maximus genome assembly, chromosome: 14	58.1	59	81.356	0.000725
LR697111.1	Chanos chanos genome assembly, chromosome: 6	59	216	66.667	0.000208
LR131916.1	Cottoperca gobio genome assembly, chromosome: 1	104	220	73.636	5.57E-18
LR736840.1	Pecten maximus genome assembly, chromosome: 3	66.2	230	70.87	1.40E-06
LR584430.1	Salmo trutta genome assembly, chromosome: 7	59	211	67.299	0.000208
LR778261.1	Coregonus sp. 'balchen' genome assembly, chromosome: 9	57.2	208	69.231	0.000725
LR633949.1	Gadus morhua genome assembly, chromosome: 7	68	215	69.302	4.01E-07
LR812512.1	Danio aesculapii genome assembly, chromosome: 7	60.8	215	72.093	5.95E-05
LR738551.1	Neostethus bicornis genome assembly, chromosome: 11	64.4	143	73.427	4.89E-06
LR597458.1	Sphaeramia orbicularis genome assembly, chromosome: 1	59.9	131	70.229	0.000208
CP027285.1	Lateolabrax maculatus chromosome Lm24	67.1	99	74.747	1.40E-06
LR990268.1	Aricia agestis genome assembly, chromosome: 12	58.1	79	75.949	0.000725
XM_034133537.1	PREDICTED: Trematomus bernacchii uncharacterized protein LOC793007 homolog (zgc:162331), transcript variant X4, mRNA	154	211	76.303	3.51E-33
XM_034133536.1	PREDICTED: Trematomus bernacchii uncharacterized protein LOC793007 homolog (zgc:162331), transcript variant X3, mRNA	154	211	76.303	3.51E-33
XM_034133535.1	PREDICTED: Trematomus bernacchii uncharacterized protein LOC793007 homolog (zgc:162331), transcript variant X2, mRNA	154	211	76.303	3.51E-33
XM_034133534.1	PREDICTED: Trematomus bernacchii uncharacterized protein LOC793007 homolog (zgc:162331), transcript variant X1, mRNA	154	211	76.303	3.51E-33
XM_034225939.1	PREDICTED: Gymnodraco acuticeps uncharacterized protein LOC793007 homolog (zgc:162331), mRNA	150	208	75.962	4.28E-32
LR899861.1	Darwinula stevensoni	58.1	129	69.767	0.000725
LR899861.1	Darwinula stevensoni	63.5	127	70.866	1.71E-05
XM_038360266.1	PREDICTED: Zerene cesonia activated Cdc42 kinase-like (LOC119835447), mRNA	67.1	89	76.404	1.40E-06
CP032597.1	Lateolabrax maculatus linkage group 22 sequence	59	77	76.623	0.000208
CP032605.1	Lateolabrax maculatus linkage group 8 sequence	59	74	78.378	0.000208
CP027276.1	Lateolabrax maculatus chromosome Lm15	59	74	78.378	0.000208
LR812081.1	Danio rerio genome assembly, chromosome: 19	63.5	209	67.464	1.71E-05

CU633204.17	Zebrafish DNA sequence from clone CH73-80E16 in linkage group 19, complete sequence	63.5	209	67.464	1.71E-05
LR584432.1	Salmo trutta genome assembly, chromosome: 30	80.6	179	69.832	6.36E-11
LR131992.1	Gouania willdenowi genome assembly, chromosome: 16	59.9	125	70.4	0.000208
LR990668.1	Xestia xanthographa genome assembly, chromosome: 27	58.1	174	67.241	0.000725
LR597479.1	Sphaeramia orbicularis genome assembly, chromosome: 22	57.2	164	67.683	0.000725
XM_034133537.1	PREDICTED: Trematomus bernacchii uncharacterized protein LOC793007 homolog (zgc:162331), transcript variant X4, mRNA	143	205	75.61	6.35E-30
XM_034133536.1	PREDICTED: Trematomus bernacchii uncharacterized protein LOC793007 homolog (zgc:162331), transcript variant X3, mRNA	143	205	75.61	6.35E-30
XM_034133535.1	PREDICTED: Trematomus bernacchii uncharacterized protein LOC793007 homolog (zgc:162331), transcript variant X2, mRNA	143	205	75.61	6.35E-30
XM_034133534.1	PREDICTED: Trematomus bernacchii uncharacterized protein LOC793007 homolog (zgc:162331), transcript variant X1, mRNA	143	205	75.61	6.35E-30
XM_034225939.1	PREDICTED: Gymnodraco acuticeps uncharacterized protein LOC793007 homolog (zgc:162331), mRNA	140	202	75.248	7.74E-29
LR697116.1	Chanos chanos genome assembly, chromosome: 11	77.9	202	69.802	7.74E-10
LR597469.1	Sphaeramia orbicularis genome assembly, chromosome: 12	59.9	210	70.476	0.000208
LR584416.1	Salmo trutta genome assembly, chromosome: 3	101	196	71.429	6.79E-17
LR697110.1	Chanos chanos genome assembly, chromosome: 5	79.7	196	68.878	2.22E-10
AC091616.2	Rattus norvegicus strain Brown Norway clone RP31-153J8, complete sequence	59.9	199	68.844	0.000208
LR744060.1	Scyliorhinus canicula genome assembly, chromosome: 31	68	171	70.175	4.01E-07
LR584432.1	Salmo trutta genome assembly, chromosome: 30	84.2	201	69.154	5.22E-12
CR382137.2	Debaryomyces hansenii CBS767 chromosome E complete sequence	62.6	119	71.429	1.71E-05
XM_459577.1	Debaryomyces hansenii CBS767 DEHA2E05852p (DEHA2E05852g), partial mRNA	62.6	119	71.429	1.71E-05
LR722980.1	Thalassophryne amazonica genome assembly, chromosome: 15	61.7	219	68.493	5.95E-05
LR722978.1	Thalassophryne amazonica genome assembly, chromosome: 13	61.7	196	68.878	5.95E-05
LR131928.1	Cottoperca gobio genome assembly, chromosome: 20	96.9	167	73.653	8.27E-16
XM_028684159.1	Plasmodium sp. gorilla clade G2 conserved Plasmodium protein, unknown function (PADL01_0312400), partial mRNA	58.1	171	69.591	0.000725
LT969412.1	Plasmodium sp. gorilla clade G2 genome assembly, chromosome: 3	58.1	171	69.591	0.000725
LR812517.1	Danio aesculapii genome assembly, chromosome: 3	77.9	196	72.959	7.74E-10
LR584416.1	Salmo trutta genome assembly, chromosome: 3	81.5	183	73.224	6.36E-11
XM_001524819.1	Lodderomyces elongisporus NRRL YB-4239 conserved hypothetical protein (LELG_03901) partial mRNA	64.4	145	69.655	4.89E-06
AP018151.1	Caenorhabditis sp. 34 TK-2017 DNA. chromosome 1, nearly complete genome	59	141	69.504	0.000208
LR584417.1	Salmo trutta genome assembly, chromosome: 37	75.2	182	72.527	2.70E-09
CP002659.1	Sphaerochaeta coccoides DSM 17374, complete genome	57.2	66	78.788	0.000725
LT594624.1	Plasmodium malariae genome assembly, chromosome: 3	72.5	194	68.557	3.29E-08
LR131466.1	Plasmodium falciparum genome assembly, chromosome: 1	57.2	186	66.667	0.000725

LR597458.1	Sphaeramia orbicularis genome assembly, chromosome: 1	103	182	74.176	1.94E-17
LR131928.1	Cottoperca gobio genome assembly, chromosome: 20	100	179	73.184	6.79E-17
LN714486.1	TPA_asm: Neospora caninum Liverpool, chromosome chrXI, complete genome	72.5	177	68.927	3.29E-08
XM_003885574.1	Neospora caninum Liverpool conserved hypothetical protein (NCLIV_060200), partial mRNA	72.5	177	68.927	3.29E-08
FR823392.1	Neospora caninum Liverpool complete genome, chromosome XI	72.5	177	68.927	3.29E-08
LR584432.1	Salmo trutta genome assembly, chromosome: 30	97.8	171	72.515	8.27E-16
LR597467.1	Sphaeramia orbicularis genome assembly, chromosome: 10	63.5	182	67.582	1.71E-05
OE840970.1	5_Tge_b3v08	57.2	198	69.192	0.000725
LR597478.1	Sphaeramia orbicularis genome assembly, chromosome: 21	80.6	179	69.832	6.36E-11
XM_023483289.1	PREDICTED: Eurytemora affinis uncharacterized LOC111709561 (LOC111709561), mRNA	63.5	98	80.612	1.71E-05
LR812070.1	Danio rerio genome assembly, chromosome: 8	57.2	181	71.271	0.000725
CP026255.1	Scophthalmus maximus chromosome 13	59	181	70.166	0.000208
CR385022.10	Zebrafish DNA sequence from clone CH211-63C12 in linkage group 23, complete sequence	58.1	173	73.988	0.000725
LR597458.1	Sphaeramia orbicularis genome assembly, chromosome: 1	71.6	145	71.034	3.29E-08
LR131999.1	Gouania willdenowi genome assembly, chromosome: 13	69.8	172	69.186	1.15E-07
LR899861.1	Darwinula stevensoni	59	132	69.697	0.000208
LR597457.1	Salarias fasciatus genome assembly, chromosome: 23	59.9	176	71.023	0.000208
LR584432.1	Salmo trutta genome assembly, chromosome: 30	88.7	161	72.05	4.28E-13
XM_027158426.1	PREDICTED: Tachysurus fulvidraco splicing factor U2af large subunit A-like (LOC113650229), mRNA	60.8	143	69.231	5.95E-05
LR131992.1	Gouania willdenowi genome assembly, chromosome: 16	72.5	142	71.127	3.29E-08
LR131928.1	Cottoperca gobio genome assembly, chromosome: 20	97.8	170	73.529	8.27E-16
LR597465.1	Sphaeramia orbicularis genome assembly, chromosome: 8	60.8	184	68.478	5.95E-05
OD567462.1	4_Tbi_b3v08	59	177	68.362	0.000208
LR812496.1	Danio aesculapii genome assembly, chromosome: 17	57.2	167	71.257	0.000725
LR131916.1	Cottoperca gobio genome assembly, chromosome: 1	75.2	171	74.269	2.70E-09
LR597464.1	Sphaeramia orbicularis genome assembly, chromosome: 7	85.1	166	71.687	5.22E-12
LR744060.1	Scyliorhinus canicula genome assembly, chromosome: 31	58.1	168	69.048	0.000725
LR812584.1	Danio rerio strain Nadia (NA) genome assembly, chromosome: 16	60.8	165	72.121	5.95E-05
CR382137.2	Debaryomyces hansenii CBS767 chromosome E complete sequence	60.8	128	70.312	5.95E-05
XM_459577.1	Debaryomyces hansenii CBS767 DEHA2E05852p (DEHA2E05852g), partial mRNA	60.8	128	70.312	5.95E-05
LR738542.1	Neostethus bicornis genome assembly, chromosome: 1	74.3	158	70.253	9.44E-09
XM_001524819.1	Lodderomyces elongisporus NRRL YB-4239 conserved hypothetical protein (LELG_03901) partial mRNA	65.3	148	69.595	4.89E-06
AP018151.1	Caenorhabditis sp. 34 TK-2017 DNA. chromosome 1, nearly complete genome	61.7	143	69.93	5.95E-05
LR736844.1	Pecten maximus genome assembly, chromosome: 7	71.6	171	71.345	3.29E-08
LR812587.1	Danio rerio strain Nadia (NA) genome assembly, chromosome: 19	60.8	151	70.199	5.95E-05

			LR129761.1	Plasmodium yoelii killicki strain 193L genome assembly, chromosome: 6	68.9	95	75.789	4.01E-07
			LS423616.1	Anas platyrhynchos genome assembly, chromosome: 6	65.3	150	73.333	4.89E-06
			OB787669.1	Cyprideis torosa	57.2	103	75.728	0.000725
			LR597464.1	Sphaeramia orbicularis genome assembly, chromosome: 7	77.9	140	72.143	7.74E-10
			LR597479.1	Sphaeramia orbicularis genome assembly, chromosome: 22	57.2	144	68.75	0.000725
			XM_009033279.1	Helobdella robusta hypothetical protein partial mRNA	59	107	71.963	0.000208
			LR597458.1	Sphaeramia orbicularis genome assembly, chromosome: 1	61.7	124	70.968	5.95E-05
			XM_034225939.1	PREDICTED: Gymnodraco acuticeps uncharacterized protein LOC793007 homolog (zgc:162331), mRNA	102	136	76.471	1.94E-17
			XM_038360266.1	PREDICTED: Zerene cesonia activated Cdc42 kinase-like (LOC119835447), mRNA	70.7	106	74.528	1.15E-07
			LR584443.1	Salmo trutta genome assembly, chromosome: 40	64.4	125	72.8	4.89E-06
			XM_034133537.1	PREDICTED: Trematomus bernacchii uncharacterized protein LOC793007 homolog (zgc:162331), transcript variant X4, mRNA	104	130	77.692	5.57E-18
			XM_034133536.1	PREDICTED: Trematomus bernacchii uncharacterized protein LOC793007 homolog (zgc:162331), transcript variant X3, mRNA	104	130	77.692	5.57E-18
			XM_034133535.1	PREDICTED: Trematomus bernacchii uncharacterized protein LOC793007 homolog (zgc:162331), transcript variant X2, mRNA	104	130	77.692	5.57E-18
			XM_034133534.1	PREDICTED: Trematomus bernacchii uncharacterized protein LOC793007 homolog (zgc:162331), transcript variant X1, mRNA	104	130	77.692	5.57E-18
			XM_001527872.1	Lodderomyces elongisporus NRRL YB-4239 hypothetical protein (LELG_00442) partial mRNA	62.6	89	75.281	1.71E-05
			XM_034225939.1	PREDICTED: Gymnodraco acuticeps uncharacterized protein LOC793007 homolog (zgc:162331), mRNA	92.4	118	77.119	3.52E-14
			LT594509.1	Plasmodium ovale genome assembly, chromosome: 5	58.1	124	70.161	0.000725
			LR129761.1	Plasmodium yoelii killicki strain 193L genome assembly, chromosome: 6	61.7	101	73.267	5.95E-05
			LT594509.1	Plasmodium ovale genome assembly, chromosome: 5	58.1	118	71.186	0.000725
			LR129761.1	Plasmodium yoelii killicki strain 193L genome assembly, chromosome: 6	76.1	111	75.676	2.70E-09
			LR597458.1	Sphaeramia orbicularis genome assembly, chromosome: 1	68	107	73.832	4.01E-07
			LR584432.1	Salmo trutta genome assembly, chromosome: 30	64.4	105	73.333	4.89E-06
			LR597477.1	Sphaeramia orbicularis genome assembly, chromosome: 20	69.8	78	79.487	1.15E-07
			LR597477.1	Sphaeramia orbicularis genome assembly, chromosome: 20	61.7	71	78.873	5.95E-05
			LR597458.1	Sphaeramia orbicularis genome assembly, chromosome: 1	63.5	95	74.737	1.71E-05
			CP032597.1	Lateolabrax maculatus linkage group 22 sequence	59.9	65	80	0.000208
			XM_024887284.1	Hyaloscypha bicolor E uncharacterized protein (K444DRAFT_667332), mRNA	58.1	73	78.082	0.000725
			LT841386.1	Plasmodium cynomolgi genome assembly, chromosome: 8	61.7	66	80.303	5.95E-05
			XM_001527872.1	Lodderomyces elongisporus NRRL YB-4239 hypothetical protein (LELG_00442) partial mRNA	68.9	85	77.647	4.01E-07
			CP024761.1	Hyphopichia burtonii isolate makgeolli chromosome 3	59	84	76.19	0.000208
NPR_8	132	ycf1 (SSC)	NA	NA	NA	NA	NA	NA

Supplementary table 2. Eight NPR sequences.

Name	Sequence
NPR_1	GGGGGGGGAAAGTATACAATATTTCCCCGAGGAGGTTTGAGTAAACTCCCGTTCTAATTCAAAAGAACGGGACTTTCGTTTTAG<br>GAAAGTCGTTTTGATTTTATGATTTATGAAATAAAATAATTCGATTTCGATAGGGTTATATAAAAAACTTCTATAGTATGAAAGG<br>GCCATTTGGGCTAGATGGAGGTCAAATAGTATGGAACAAACAAAAGTGTATTCAACAGGTTGTCTTTTTGACAATAAGCTTGTCT<br>GTAATACTAACTCTTATGA
NPR_2	CTCACGTTCGGACATTTTGCGCACTTCACGAATGGGTTGCAAAACCATTTTTACACGGTCCCTTTTATCAACCCGCCATGGTTCGG<br>GAGAAAACGCTAGGGATTCGGGCTTTAATTTTCTTCTTTTTAAACAAGTTATTATTACCTTGAACTTTTTTTTTTTTTTTGAATTTT<br>CTGTCGCTAA
NPR_3	GTTGGTCTAGTATTACTCTTTGTAATCTTTCAAACTCCGTTTCGTCTTCGCTTCTGTTGTCGGGCTCGCGCTCCAAACTCACAGAG<br>ACAGTAGAAAAACAGAAGTTTTTTCTTAGAGTAAAATGGTATTCTTTCAAAAGAAACGCGACTTCTGAGAATAACCAAAGAGAC<br>GAACTCAATAATATTTTTTTTAATACTTTTTTA
NPR_4	CCGATTCATTATCGTTCACATATAGTTTTGAAAAATGAAAATATTTTCTATCTAACTTTTTGAGCCTAGTCTCAGGGTTTATATCA<br>CGAAAAAGGAAGTGGCCCCTGCTATTTTTCATATT
NPR_5	TCCCTTGTCTTTTCGTCTGCATTTTCGTCCACATTTTCGTCCGCATTTTCATCTGTGCTTTCGTCTGTGCTTTTGTCTGTGCTTTCGT<br>CTGTGCTTTCGTCTGTGCTTCTGTATATTCTTTTGTCTGTGCTTTCGTCTGTGCTTCTGTATATTCTTTTGTCTGTGCTTTCGTCTGT<br>GCTTCTGTATATTCTTTTGTCTGTGCTTTCGTCTGTGCTTCTGTATATTCTTTTGTCTGTGCTTTCGTCTGTGCTTCTGTATATTCTTT<br>TGTCTGTGCTTTCGTCTGTGCTTTCGTCTGTGCTTCTGTATATTCTTTTGTCTGTGCTTTTGTCTTTTTCTTCTAGGTTTAGA
NPR_6	TCGGTTAATAGGTCGAAAAATAGGTCACTTCTAAAGCCTTTACCAAGTTGCATAATAAAACCCTCCATCCTCTTTTAGGATTAAT<br>AAAAAAATTTATTTGCTATTGCAAAATAAAATTATGTATAATTATTG
NPR_7	CTATTATATCAAAGATAATAATAGTTGTCTTAAACATTTTGAAGTAAGTATCTGTTTTTGAATTTGATGGAGCTTCAAAATTATCA<br>AATTCCGGGGCATCCAGATTTTGATCCGGATTAGGCCTTTCGCCACTATTCAAATTTGATGGAGCTTCAAAATTATCAAATTTCG<br>GGGCATCCAGATTTTGATCCGGATTAGGCCTTTCGCCACAAAAAACATTGTCTTTAGTTTTACGTTCACAGACCGCGCAAGGTTC<br>TATTTTTTCAATAACTTCCCGGTTATCCTCTTCCAAGACCTCTTCTCTACCAATAACTTCCCGCGTATCTAGTTCCAAGACCTCACA<br>AGTTTGCCTCTGTAGTAGATTGCCGAACCAAGGTTTGTTCCACATCCGCGCCTCAAGTAAGCGTAGGAGTATCAAAACCGTGAAT<br>CCGGCTATGTACTTTTCAGCCGTTGTTAACTTTCTCATATTTCTCCTAAATAGAATAATTATTCTAGTTTTGCAAATAGCCAATTCT<br>TTTATC
NPR_8	ATGTGTATATGAAGTATACATGAAGTATATCCAACTAGAATATCTTTGTCAACTAATTATTA

Supplementary table 3. Simple sequence repeats in the plastome of *V. sambucifolia* f. *dageletiana*

Repeat type	Motif	Number of motif														
		3	4	5	6	7	8	9	10	11	12	13	14	15	16	17
Mono-nucleotide	A/T	-	-	-	-	111	33	33	6	8	6	1	5	3	1	2
	C/G	-	-	-	-	16	5	2	0	0	1	1	0	0	0	0
Di-nucleotide	AT	-	-	2	2	0	0	0	0	0	0	0	0	0	0	0
	TA	-	-	0	0	1	0	0	0	0	0	0	0	0	0	0
Tetra-nucleotide	AAAT/ ATTT	2	0	0	0	0	0	0	0	0	0	0	0	0	0	0
	TATT/ AATA	1	0	0	0	0	0	0	0	0	0	0	0	0	0	0
Hexa-nucleotide	CCTACC/ GGTAGG	0	1	0	0	0	0	0	0	0	0	0	0	0	0	0
	GGGGTA/ TACCCC	0	1	0	0	0	0	0	0	0	0	0	0	0	0	0
	TAGAAG/ CTTCTA	1	0	0	0	0	0	0	0	0	0	0	0	0	0	0
	TTCTAC/ GTAGAA	1	0	0	0	0	0	0	0	0	0	0	0	0	0	0

Supplementary table 4. Simple sequence repeats in the mitogenome of *V. sambucifolia* f. *dageletiana*

Repeat type	Motif	Number of repeats																
		3	4	5	6	7	8	9	10	11	12	13	14	15	16	17	18	19
Mono-nucleotide	A/T	-	-	-	-	454	196	74	44	14	7	4	3	5	3	1	1	1
	C/G	-	-	-	-	74	17	3	0	0	0	0	0	0	0	0	0	0
Di-nucleotide	AG/CT	-	-	13	1	0	0	0	0	0	0	0	0	0	0	0	0	0
	AT	-	-	15	0	0	0	2	0	1	0	0	1	0	0	0	0	0
	CA/TG	-	-	2	0	0	0	0	0	0	0	0	0	0	0	0	0	0
	GA/TC	-	-	13	1	0	0	0	0	0	0	0	0	0	0	0	0	0
	TA	-	-	13	6	1	2	1	1	0	0	0	0	0	1	0	0	0
Tri-nucleotide	AAG/CTT	-	11	0	0	0	0	0	0	0	0	0	0	0	0	0	0	0
	AAT/ATT	-	2	0	1	0	0	0	0	0	0	0	0	0	0	0	0	0
	ACA/TGT	-	3	0	0	0	0	0	0	0	0	0	0	0	0	0	0	0
	ACT/AGT	-	1	0	0	0	0	0	0	0	1	0	0	0	0	0	0	0
	AGA/TCT	-	5	0	0	0	0	0	0	0	0	0	0	0	0	0	0	0
	ATA/TAT	-	1	0	0	0	0	0	0	0	0	0	0	0	0	0	0	0
	ATG/CAT	-	2	0	0	0	0	0	0	0	0	0	0	0	0	0	0	0
	CTC/GAG	-	1	0	0	0	0	0	0	0	0	0	0	0	0	0	0	0
	CTG/CAG	-	1	0	0	0	0	0	0	0	0	0	0	0	0	0	0	0
	GAA/TTC	-	9	0	0	0	0	0	0	0	0	0	0	0	0	0	0	0
	GCT/AGC	-	0	1	0	0	0	0	0	0	0	0	0	0	0	0	0	0
	GGA/TCC	-	1	0	0	0	0	0	0	0	0	0	0	0	0	0	0	0
	TAA/TTA	-	10	1	0	0	0	0	0	0	0	0	0	0	0	0	0	0
	TAG/CTA	-	1	0	0	0	0	0	0	0	0	0	0	0	0	0	0	0
	TGA/TCA	-	1	0	0	0	0	0	0	0	0	0	0	0	0	0	0	0
	TTG/CAA	-	1	0	0	0	0	0	0	0	0	0	0	0	0	0	0	0
Tetra-nucleotide	AAAG/CTTT	18	1	0	0	0	0	0	0	0	0	0	0	0	0	0	0	0
	AAAT/ATTT	8	1	0	0	0	0	0	0	0	0	0	0	0	0	0	0	0
	AAGA/TCTT	14	2	1	0	0	0	0	0	0	0	0	0	0	0	0	0	0
	AAGC/GCTT	1	0	0	0	0	0	0	0	0	0	0	0	0	0	0	0	0
	AATA/TATT	0	1	0	0	0	0	0	0	0	0	0	0	0	0	0	0	0
	ACCT/AGGT	2	1	0	0	0	0	0	0	0	0	0	0	0	0	0	0	0
	AGAA/TTCT	16	0	0	0	0	0	0	0	0	0	0	0	0	0	0	0	0
	AGAT/ATCT	0	1	1	0	0	0	0	0	0	0	0	0	0	0	0	0	0
	AGCC/GGCT	1	0	0	0	0	0	0	0	0	0	0	0	0	0	0	0	0
	AGCT	1	0	0	0	0	0	0	0	0	0	0	0	0	0	0	0	0
	AGTG/CACT	1	0	0	0	0	0	0	0	0	0	0	0	0	0	0	0	0
	ATAA/TTAT	3	2	1	0	0	0	0	0	0	0	0	0	0	0	0	0	0
	ATAG/CTAT	3	0	0	0	0	0	0	0	0	0	0	0	0	0	0	0	0
	ATGA/TCAT	1	1	0	0	0	0	0	0	0	0	0	0	0	0	0	0	0
	ATTC/GAAT	3	0	0	0	0	0	0	0	0	0	0	0	0	0	0	0	0
	ATTG/CAAT	1	0	0	0	0	0	0	0	0	0	0	0	0	0	0	0	0
	CATT/AATG	1	0	0	0	0	0	0	0	0	0	0	0	0	0	0	0	0
	CCCT/AGGG	1	0	0	0	0	0	0	0	0	0	0	0	0	0	0	0	0
	CTGC/GCAG	1	0	0	0	0	0	0	0	0	0	0	0	0	0	0	0	0
	CTGG/CCAG	1	0	0	0	0	0	0	0	0	0	0	0	0	0	0	0	0
	CTTA/TAAG	2	0	0	0	0	0	0	0	0	0	0	0	0	0	0	0	0
	GAAA/TTTC	13	0	0	0	0	0	0	0	0	0	0	0	0	0	0	0	0
	GAAG/CTTC	1	0	0	0	0	0	0	0	0	0	0	0	0	0	0	0	0
	GATA/TATC	2	0	0	0	0	0	0	0	0	0	0	0	0	0	0	0	0
	GCAA/TTGC	2	0	0	0	0	0	0	0	0	0	0	0	0	0	0	0	0
	GTAT/ATAC	1	0	0	0	0	0	0	0	0	0	0	0	0	0	0	0	0
	TAAA/TTTA	1	0	0	0	0	0	0	0	0	0	0	0	0	0	0	0	0
	TATG/CATA	1	0	0	0	0	0	0	0	0	0	0	0	0	0	0	0	0
	TCAC/GTGA	1	0	0	0	0	0	0	0	0	0	0	0	0	0	0	0	0
	TGCA	1	0	0	0	0	0	0	0	0	0	0	0	0	0	0	0	0
	TGCT/AGCA	2	0	0	0	0	0	0	0	0	0	0	0	0	0	0	0	0
	TGGA/TCCA	1	0	0	0	0	0	0	0	0	0	0	0	0	0	0	0	0
	TTAA	1	0	0	0	0	0	0	0	0	0	0	0	0	0	0	0	0
	TTCA/TGAA	2	0	0	0	0	0	0	0	0	0	0	0	0	0	0	0	0
	TTCC/GGAA	1	0	0	0	0	0	0	0	0	0	0	0	0	0	0	0	0
Penta-nucleotide	AAAAG/CTTTT	2	0	0	0	0	0	0	0	0	0	0	0	0	0	0	0	0
	AAGAA/TTCTT	1	0	0	0	0	0	0	0	0	0	0	0	0	0	0	0	0
	AATAT/ATATT	1	0	0	0	1	0	0	0	0	0	0	0	0	0	0	0	0
	ACAAC/GTTGT	3	0	0	0	0	0	0	0	0	0	0	0	0	0	0	0	0

	ACCTT/AAGGT	1	0	0	0	0	0	0	0	0	0	0	0	0	0	0	0	0
	ACTAA/TTAGT	1	0	0	0	0	0	0	0	0	0	0	0	0	0	0	0	0
	ACTAT/ATAGT	0	1	0	0	0	0	0	0	0	0	0	0	0	0	0	0	0
	ACTCT/AGAGT	1	0	0	0	0	0	0	0	0	0	0	0	0	0	0	0	0
	AGAGA/TCTCT	1	0	0	0	0	0	0	0	0	0	0	0	0	0	0	0	0
	AGGAG/CTCCT	1	0	0	0	0	0	0	0	0	0	0	0	0	0	0	0	0
	AGTAG/CTACT	1	0	0	0	0	0	0	0	0	0	0	0	0	0	0	0	0
	AGTTT/AAACT	0	1	0	0	0	0	0	0	0	0	0	0	0	0	0	0	0
	ATAAG/CTTAT	1	0	0	0	0	0	0	0	0	0	0	0	0	0	0	0	0
	ATAGA/TCTAT	2	0	0	1	0	0	0	0	0	0	0	0	0	0	0	0	0
	ATTAG/CTAAT	1	0	0	0	0	0	0	0	0	0	0	0	0	0	0	0	0
	CCTTT/AAAGG	1	0	0	0	0	0	0	0	0	0	0	0	0	0	0	0	0
	CTTAA/TTAAG	1	0	0	0	0	0	0	0	0	0	0	0	0	0	0	0	0
	CTTTA/TAAAG	2	0	0	0	0	0	0	0	0	0	0	0	0	0	0	0	0
	GATGA/TCATC	1	0	0	0	0	0	0	0	0	0	0	0	0	0	0	0	0
	GGGAT/ATCCC	1	0	0	0	0	0	0	0	0	0	0	0	0	0	0	0	0
	TAACT/AGTTA	1	0	0	0	0	0	0	0	0	0	0	0	0	0	0	0	0
	TAAGC/GCTTA	1	0	0	0	0	0	0	0	0	0	0	0	0	0	0	0	0
	TACTA/TAGTA	0	2	0	0	0	0	0	0	0	0	0	0	0	0	0	0	0
	TATAT/ATATA	1	0	0	0	0	0	0	0	0	0	0	0	0	0	0	0	0
	TATGG/CCATA	1	0	0	0	0	0	0	0	0	0	0	0	0	0	0	0	0
	TATTA/TAATA	2	0	0	0	0	0	0	0	0	0	0	0	0	0	0	0	0
	TCTTA/TAAGA	2	0	0	0	0	0	0	0	0	0	0	0	0	0	0	0	0
	TCTTT/AAAGA	1	0	0	0	0	0	0	0	0	0	0	0	0	0	0	0	0
	TTATA/TATAA	0	1	0	0	0	0	0	0	0	0	0	0	0	0	0	0	0
	TTCTC/GAGAA	1	0	0	0	0	0	0	0	0	0	0	0	0	0	0	0	0
	TTTTA/TAAAA	1	0	0	0	0	0	0	0	0	0	0	0	0	0	0	0	0
	AAAATG/CATTTT	1	0	0	0	0	0	0	0	0	0	0	0	0	0	0	0	0
	AGAAAA/TTTTCT	1	0	0	0	0	0	0	0	0	0	0	0	0	0	0	0	0
	AGAAGT/ACTTCT	1	0	0	0	0	0	0	0	0	0	0	0	0	0	0	0	0
	AGGCTC/GAGCCT	0	1	0	0	0	0	0	0	0	0	0	0	0	0	0	0	0
	AGTTAT/ATAACT	0	1	0	0	0	0	0	0	0	0	0	0	0	0	0	0	0
	ATATAA/TTATAT	1	0	0	0	0	0	0	0	0	0	0	0	0	0	0	0	0
Hexa-nucleotide	ATTACT/AGTAAT	1	0	0	0	0	0	0	0	0	0	0	0	0	0	0	0	0
	CAAATT/AATTTG	1	0	0	0	0	0	0	0	0	0	0	0	0	0	0	0	0
	CTTGAG/CTCAAG	1	0	0	0	0	0	0	0	0	0	0	0	0	0	0	0	0
	TCCACT/AGTGGA	1	0	0	0	0	0	0	0	0	0	0	0	0	0	0	0	0
	TCCTTA/TAAGGA	1	0	0	0	0	0	0	0	0	0	0	0	0	0	0	0	0
	TCTCTT/AAGAGA	1	0	0	0	0	0	0	0	0	0	0	0	0	0	0	0	0
	TCTGCT/AGCAGA	1	0	0	0	0	0	0	0	0	0	0	0	0	0	0	0	0

Supplementary table 5. Information of next generation sequencing data

Library type [a]	Raw reads			Reads after trimming		
	Total read bases (bp)	Total reads	Sequence Length (bp)	Total read bases (bp)	Total reads	Sequence Length (bp)
MP	3,152,082,418	20,874,718	151	2,556,315,822	17,888,600	50-151
PE	6,805,768,862	45,129,454	36-151 [b]	5,251,686,554	38,045,130	50-151
PE	12,188,335,252	80,717,452	151	10,727,252,260	73,012,430	50-151

[a] : MP (mate-pair), PE (paired-end)

[b] : Adapter trimming using Scythe by supplier

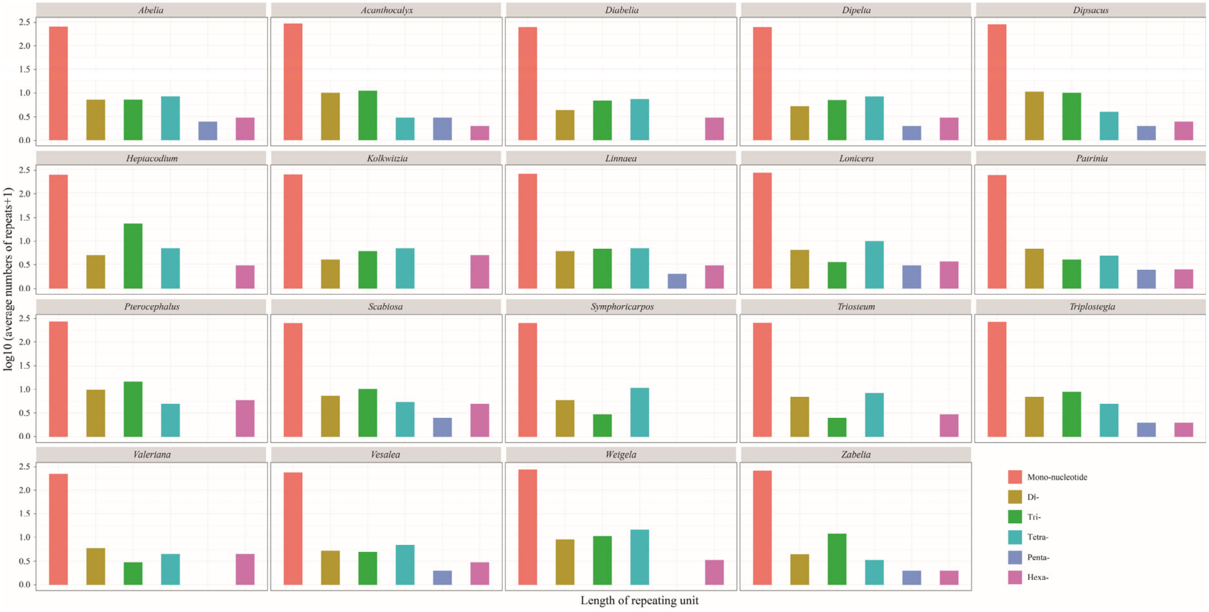

Supplementary figure 1. Simple sequence repeats at the genus level in Caprifoliaceae. Different color refers to different length of motif.

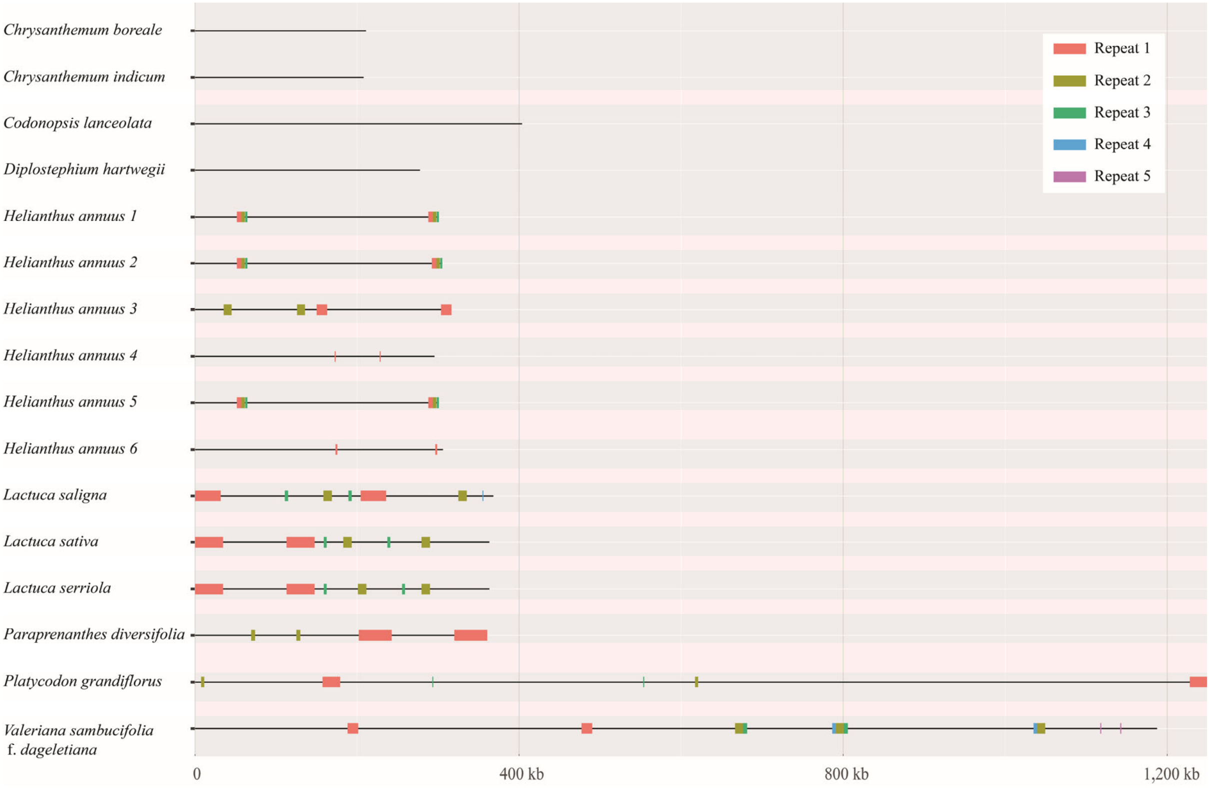

Supplementary figure 2. The identical repeats in mitogenome of *V. sambucifolia* f. *dageletiana* compared with those of Asterales

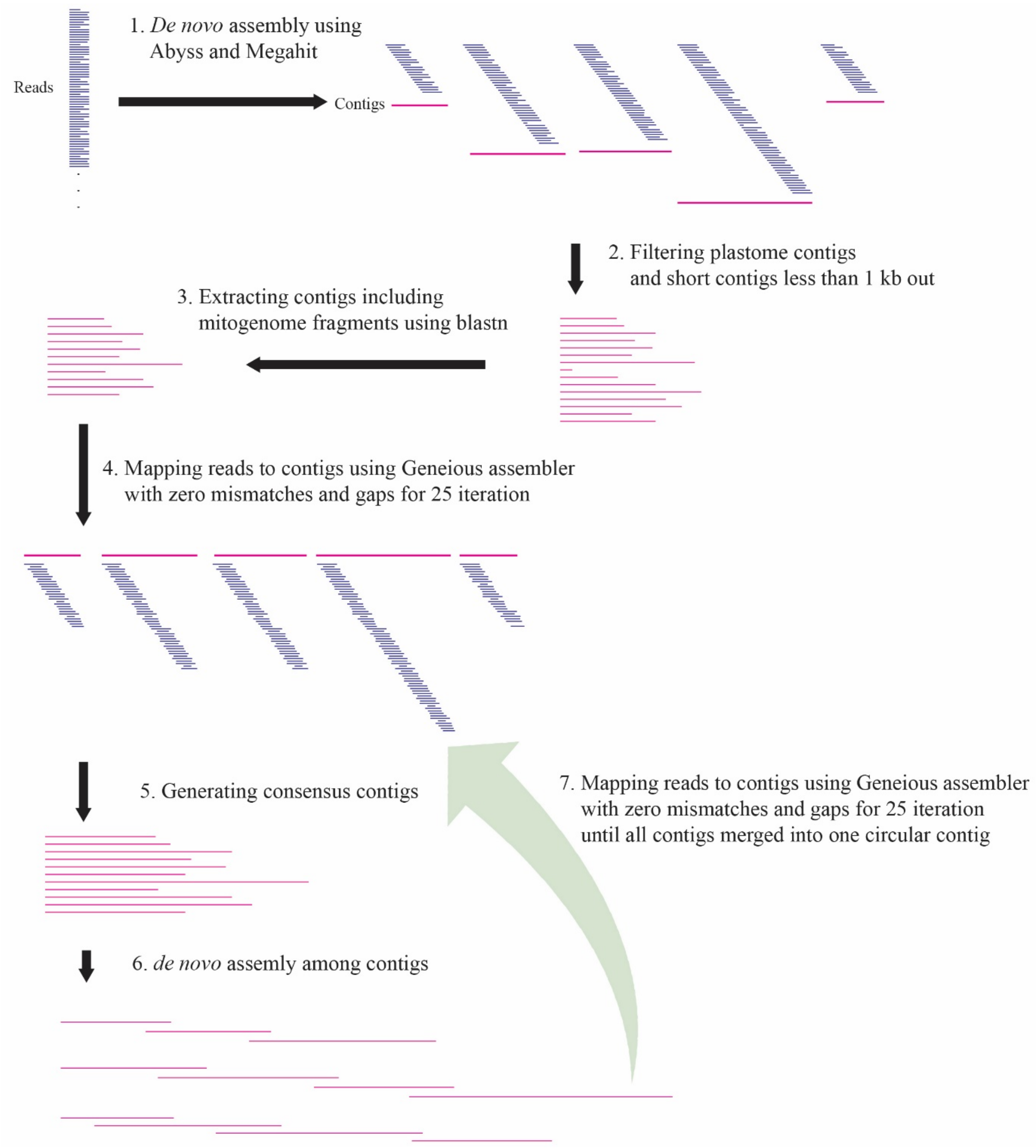

Supplementary figure 3. Assembly strategy for mitogenome of *V. sambucifolia* f. *dageletiana*
